# Genome-wide identification of the auxin response factor gene family in Cicer arietinum

**DOI:** 10.1186/s12864-018-4695-9

**Published:** 2018-04-27

**Authors:** Jose V. Die, Juan Gil, Teresa Millan

**Affiliations:** Department of Genetics, ETSIAM, University of Córdoba, Córdoba, Spain

**Keywords:** ARF, Bioinformatics, Chickpea, Gene duplication, Gene expression, RT-qPCR, Rstat

## Abstract

**Background:**

Auxin Response Factors act as critical components of the auxin-signaling pathway by regulating the transcription of auxin-responsive genes. The release of the chickpea reference genome provides an opportunity to identify and characterize the ARF gene family in this important legume by a data mining coupled by comparative genomics approaches.

**Results:**

We performed a comprehensive characterization and analysis of 24 ARF genes in the chickpea reference genome. Comparative phylogenetic analysis of the ARF from chickpea, *Medicago* and *Arabidopsis* suggests that recent duplications have played a very limited role in the expansion of the ARF chickpea family. Gene structure analysis based on exon-intron organization provides additional evidence to support the evolutionary relationship among the ARF members. Conserved motif analysis shows that most of the proteins fit into the canonical ARF structure model, but 9 proteins lack or have a truncated dimerization domain. The mechanisms underlying the diversification of the ARF gene family are based on duplications, variations in domain organization and alternative splicing. Concerning duplications, segmental, but not tandem duplications, have contributed to the expansion of the gene family. Moreover, the duplicated pair genes have evolved mainly under the influence of purifying selection pressure with restricted functional divergence. Expression profiles responding to various environmental stimuli show a close relationship between tissue and expression patterns. Promoter sequence analysis reveals an enrichment of several *cis*-regulatory elements related to symbiosis, and modulation of plant gene expression during the interaction with microbes.

**Conclusions:**

In conclusion, this study provides a comprehensive overview of the ARF gene family in chickpea. Globally, our data supports that auxin signaling pathway regulates a wide range of physiological processes and stress responses. Our findings could further provide new insights into the complexity of the regulation of ARF at the transcription level that may be useful to develop rational chickpea breeding strategies to improve development or stress responses. Our study also provides a foundation for comparative genomic analyses and a framework to trace the dynamic evolution of ARF genes on a large time-scale within the legume family.

**Electronic supplementary material:**

The online version of this article (10.1186/s12864-018-4695-9) contains supplementary material, which is available to authorized users.

## Background

The plant hormone auxin (indole-3-acetic acid) is a key regulator of virtually every aspect of plant growth and development. Most of these processes are initiated or mediated through auxin-regulated gene expression, which in turn is controlled by proteins belonging three gene families: receptors (F-box), repressors (Auxin/Indole-3-Acetic Acid; Aux/IAA) and auxin response factor proteins (ARF). ARF represent the core of auxin signaling [1]. In the last few years, a considerable amount of new information has appeared on the regulation of ARF gene expression, target genes controlled by ARF, and the mechanisms by which ARF regulate those target genes. According to transient assays and sequence analysis, ARF proteins are divided into transcriptional activators and repressors [2]. ARF activators are transcription factors that mediate auxin-dependent transcriptional regulation by binding to auxin-response elements in the promoters of auxin-inducible genes in a dose-dependent manner [3]. In the absence of auxin, Aux/IAA proteins prevent ARF-mediated transcription by forming heterodimers with ARF activators [4, 5]. In the presence of auxin, Aux/IAA proteins are targeted to the 26S proteasome, which can be hypothesized to release interacting ARF activators from inhibition [6]. Contrary to ARF activators, ARF repressors have very limited interactions with other ARF and Aux/IAA proteins [5]. Though some auxin responses occur throughout the plant, others depend on the actual developmental context, conferring the tissue-specific response to auxin. Those responses involve the action of specific pairs of ARF and Aux/IAA proteins [7, 8]. Concerning the specificity, the domain architecture of ARF proteins plays an important role. Most ARF consist of an N-terminal DNA-binding domain (DBD), a variable middle region (MR) and a carboy-terminal dimerization domain (CTD, domains III and IV). The MR confers transcriptional activation or repression depending on its amino acid composition. Thus, the MR enriched for glutamine residues function as activation regions, while MRs serine-rich, serine and proline-rich, and serine and glycine-rich function as repression regions in *Arabidopsis thaliana* [9, 10]. Domains III and IV are essential for the heterodimerization between ARF and Aux/IAA proteins [11, 12]. These domains are also known to facilitate homodimerization, which appears to be required in some cases for the efficient binding of ARF to DNA [10].

In recent years, many of the major crops have been sequenced. Crop genome sequences, even at the current level of completeness have had a major impact on crop research or improvement in a relatively short time [13]. Chickpea (*Cicer arietinum* L.) is globally the second most important grain legume [14]. Although the chickpea yield potential has increased over the last years, the production is constrained by several major abiotic (drought, heat, high salinity) and biotic stresses (fusarium wilt, ascochyta blight) [15, 16]. Genomic resources represent the starting point for understanding the unique traits present in a given crop and are also tools for implementation of molecular breeding for the development of improved varieties [17]. Until recently, lack of information on legume genomes traditionally restricted the genome-wide survey of genes in response to the environment or stresses. Fortunately, the genome sequence of chickpea has become available in the last few years [18, 19]. Chickpea genome sequences provide an unprecedented resource, which can be exploited in numerous ways.

As a central role of the auxin-signaling pathway, the ARF multigene family is present in all major divisions of land plants [6]. The ARF family has been characterized in both annual herbaceous plants and woody perennials. Gene member numbers are variable between species ranging from 18 in peach [20] to 51 members in soybean [21]. Considering the important role of ARF family members as regulators of plant growth and developmental processes in other plant species, it is important to explore this gene family in chickpea. In this work, we provide comprehensive information on the genomic structures, chromosomal locations, sequences homology, evolutionary duplication history, *cis*-regulatory elements and expression profiles of 24 ARF genes in *C. arietinum*.

## Methods

### Genome-wide survey of ARF genes in *C. arietinum*

Comprehensive identification of *C. arietinum* ARF gene family members was achieved using *Medicago truncatula* ARF proteins. The *M. truncatula* ARF protein sequences were downloaded from the Phytozome v12.0 database (http://www.phytozome.net) and used as queries in BLASTP searches [22] to identify the corresponding ARF gene members in the chickpea proteome using a cut-off of 30% identity, 30% query coverage and e-value < 1.0E-10. For validation, we also used *Arabidopsis* ARF proteins as queries following the same procedure. The hidden Markov model (HMM) profiles of the ARF gene family [Pfam 02309: AUX/IAA family; Pfam 06507: ARF (AUX_RESP); Pfam 02362: B3 DNA binding domain (B3)] were used to confirm the identity of the candidate chickpea ARF genes. The domains of all obtained ARF were further confirmed as well by using the NCBI Conserved Domain Database (CDD, https://www.ncbi.nlm.nih.gov/cdd/) and e-value of 0.01 [23]. For exhaustive identification of divergent chickpea gene family members, we used the chickpea ARF proteins as queries in BLASTP searches against the chickpea proteome. In order to check for any possible non-predicted gene, we run tBLASTn searches against the *C. arietinum* CDC Frontier genome assembly v1 (ASM33114v1 assembly, https://www.ncbi.nlm.nih.gov/assembly/GCF_000331145.1/). All that process enabled us to obtain 45 unique ARF protein sequences. Using one gene model per locus, we identified 24 *C. arietinum* non-redundant ARF genes. Information on chromosomal location, locus ID, amino acid length, molecular weight and number of exons was retrieved from the NCBI using custom R scripts. The Compute pI tool on the ExPASy proteomics server database (http://web.expasy.org/compute_pi/) was used to predict the theoretical isoelectric point (pI) of each CaARF protein.

### Sequence alignment, prediction of amino-acid content, and protein classification

Multiple sequence alignments were conducted on the full length of the 24 ARF protein sequences using the default parameters of the MUSCLE program [24]. Amino acid content of the MR domain in CpARF was calculated using the ‘Biostrings’ R package (version 2.42; [25]). The classification of CpARF was based on the respective amino acid content [Domains with CTD: Glutamine/serine/leucine (QSL)-rich MR; Repressor with a carboxyl terminal domain (CTD); Serine/proline/glycine/leucine (SPGL)-rich MR; Repressor without CTD: Glycine-rich MR].

### Phylogenetic analysis and gene structure

The evolutionary history was inferred using the Neighbor-Joining method [26]. The bootstrap consensus tree inferred from 1000 replicates [27]. The evolutionary distances were computed using the Poisson correction method [28] and are in the units of the number of amino acid substitutions per site. Evolutionary analyses were conducted in MEGA5 [29]. The exon/intron structure of the chickpea ARF genes was based on the genome and coding sequences and was identified using the GSDS software (http://gsds.cbi.pku.edu.cn/; [30]).

### Gene duplication analysis

Duplication analysis for CaARF genes was performed using Plant Genome Duplication Database (PGDD; http://chibba.agtec.uga.edu/duplication/; [31]). Circoletto tool was used to determine and plot sequence similarity [32]. To define a tandem cluster, the following parameters were established: a cluster should contain at least two genes; a sliding window size should be < 250 kb [33]. The number of nonsynonymous substitutions per nonsynonymous site (Ka) and synonymous substitution per synonymous site (Ks) values were extracted from PGDD. The Ks values obtained for each gene pair were then translated into divergence time in millions of years assuming a rate of 6.1 × 10–9 substitutions per site per year for eudicots [34]. The divergence time (T) was calculated as T = Ks/2GAMMA (GAMMA = 6.1 × 10–9; [35]).

### In silico expression analysis

The coding sequences of ARF genes were employed to query the NCBI chickpea ESTs. Searching parameters were set as follows: megablast, identity > 95%, length > 200 bp and E-value < 10–10.

### Plant material

Plant material and treatments have been described in detail elsewhere [36]. Briefly, chickpea plants were grown in a growth chamber (12 h of light at 25 °C and 12 h of dark at 22 °C) until the moment that stress treatments started. Two weeks after flowering, some plants were exposed to cold and drought treatments. For cold treatment, a pool was exposed to a cycle of 12 h day/12 h night at temperatures of 25 °C and 5 °C, respectively. Leaves and flowers from the susceptible ICC4918 genotype (desi) were collected after the seventh night at 5 °C. Another pool was exposed to drought conditions by allowing the 5–10% loss of their water content per day. Leaves and stems from the susceptible ILC72 genotype (kabuli) were collected when the treatment pots lost 50% of their water content. For the salinity stress, 18-day-old plants were irrigated with Hoagland’s nutrient medium with 150 mM NaCl (pH 6.5). Leaf, stem and root tissues from the susceptible ICCV2 genotype (kabuli) were collected 24 h after treatment. Two ascochyta blight differential germplasm lines (WR315 susceptible, and ILC3279 resistant; desi and kabuli, respectively) were inoculated with a highly virulent isolate of *Ascochyta rabiei*. Spore suspensions at concentrations of 5 × 10^5^ were prepared from 14-day-old fungal cultures that were grown on V8 agar at 20 °C and 12 h light/dark. Inoculations were performed on two-week-old plants by spraying approximately 5 ml of the spore suspension on each plant. The inoculated plants were incubated in the dark at 20 °C and 100% continuous relative humidity for 24 h to facilitate infection. Plants were then placed in a growth chamber that was set to cycle at 12 h day (20 °C) and 12 h night (16 °C) and 100% relative humidity. Leaf tissues from inoculated plants were collected 72 h after inoculation. Control roots and leaves were separately harvested from 19-day-old plants grown in the growth chamber in 12 h day (25 °C) /12 h night (22 °C). All tissues were immediately frozen in liquid nitrogen and stored at − 80 °C until RNA extraction. Each sample was made by a pool of at least five plants. Two biological repetitions were performed for each treatment.

### RNA extractions and quality controls

Total RNA was isolated using the TRIZOL reagent (Invitrogen, CA, USA) according to the manufacturer’s protocol. RNA concentration was determined by measuring the optical density at 260 nm using a NanoDrop ND-1000 spectrophotometer (Nanodrop Technologies, USA). RNA quality was assessed by combining information from several control steps. First, purity was inferred from the absorption ratios using the NanoDrop. Only the RNA samples with A260/A280 ratio between 1.75 and 2.1 and A260/A230 greater than 2.0 were used in the analysis. Then, we amplified segments of the 5′ and 3′ regions of a malate oxidoreductase gene across the cDNA samples by qPCR (as described below) to infer the integrity of the total RNA. After NanoDrop measurements and integrity checking, all RNA samples were adjusted to the same concentration, measured and adjusted again to homogenize RNA input in the subsequent reverse-transcription reactions.

### First strand cDNA synthesis and quality controls

Total RNA (1 μg) was reverse-transcribed using the QuantiTec Reverse Transcription Kit (Qiagen, Hilden, Germany), according to the manufacturer’s instructions. We tested for presence of genomic DNA contamination (gDNA) by performing minus RT (−RT) controls, containing all components (including template RNA) except the reverse transcriptase. As a positive control, a quantity equivalent to that of the cDNA used as a template in the subsequent qPCRs amplification (i.e. 10 ng of genomic DNA) was amplified using a primer pair designed from an exon of a tubulin sequence (GR913042). The cDNA samples were considered to be suitable for further analysis because no amplification was detected in any -RT control after 40 cycles. The cDNAs were diluted to a final volume of 100 μl. The efficiency of cDNA synthesis, which in turn is dependent on the intactness of mRNA (RNA integrity) was examined using a 3′:5′ amplification ratio assessment [37] to amplify cDNA fragments in the 5′ (81 bp) and 3′ (80 bp) regions of a malate oxidoreductase gene (MOR; AJ404642). The fragments are 1671 and 450 bp, respectively, from the 3′ end of the cDNA. The primer sequences are MOR_5’F, 5’-CGACCGTTGTCTGATTTTGTGA-3′; MOR_5’R, 5’-GGCCATTTTCAGAACCCCTAA-3′; and MOR_3’F, 5’-GCTTCGAGCAGCAGTTGAAGAA-3′; and MOR_3’R, 5’-CTTTTGACATGTGTGCAAGTT-3′. The 3′:5′ amplification ratio of the MOR cDNA fragments was calculated using the comparative Cq method [38]. The average ratio was 1.51 ± 0.11. All ratios were < 3.8-fold. Only if ratios were > 4.4-fold would RNA quality be deemed inadequate [39]. Therefore, the cDNAs were judged to be suitable for qPCR analysis.

### Primer design and quality controls

ARF primers were designed using the following criteria: Tm of 60 ± 1 °C and PCR amplicon lengths of 70–95 bp, yielding primer sequences with lengths of 19–22 nucleotides and GC contents of 45–60%. For primer design improvement, amplicon sequences were checked with the nucleic acid-folding software MFOLD version 3.4 software [40]. Potential formation of secondary structures were evaluated with default settings of minimal free energy, 50 mM Na^+^, 3 mM Mg^2+^, and an annealing temperature of 60 °C. We chose primers that would yield amplicons with minimal folding structures and melting temperatures that would not hamper annealing. Designed primers were synthesized by Integrated DNA Technologies (Leuven, Belgium).

### Real-time qPCR assays and normalization

Real-time qPCR reactions (RT-qPCR) were carried out in a CFX Connect Real-Time PCR Detection System thermal cycler (Bio-Rad, Hercules, CA, USA) using iTaq Universal SYBR Green supermix (Bio-Rad) to monitor dsDNA synthesis. Reactions contained 1.5 μl of the diluted cDNA as a template and 0.2 μM of each primer in a total volume reaction of 10 μl. The following standard thermal profile was used for all PCRs: polymerase activation (95 °C for 3 min), amplification and quantification cycles repeated 40 times (95 °C for 3 s, 60 °C for 30 s). The specificity of the primer pairs was checked by melting-curve analysis performed by the PCR machine after 40 amplification cycles (60 to 95 °C). Fluorescence was analyzed using CFX Manager Software v2.1 (Bio-Rad). All amplification plots were analyzed using a base line threshold of 100 relative fluorescence units (RFU) to obtain Cq (quantification cycle) values for each gene-cDNA combination. To normalization of data, we evaluated the stable expression of four reference genes in our dataset. Three candidates encoding a protein phospatase protein (*PP2A*), pentatricopeptide repeat-containing protein (PPR) and ubiquitin-like protein (*UBQ*) were selected based on previous reports [36]. We also tested the expression of a transcription factor initiation IIA (*TFIIA*) whose homolog in pea was one of the most stable genes under a variety of conditions [41]. Two programs were used to determine which reference genes were best suited for transcript normalization. We first used the statistical algorithm geNorm [42]. In a second approach, the coefficient of variation of normalized relative expression levels was calculated according to the formulas described in the qBase software [43]. The results indicated that *PP2A* and *TFIIA* were the most stable references in our dataset with values very inside the optimal range for heterogeneous sample panels (*M* and CV values lower than 1 and 0.5 respectively according to Hellemans et al. (2007) [43] (Additional file 1: Figure S1). Therefore, the expression of each target ARF gene was normalized to the geometric average of *PP2A* and *TFIIA*. The overall mean real-time qPCR amplification efficiency of each primer pair (E) was estimated from linear regression analysis and the eq. (1 + E) = 10^slope^ implemented in the LinReg software [44]. Finally, the expression levels of the chickpea ARF genes were calculated using the advanced relative quantification model with efficiency correction, multiple reference gene normalization and the use of error propagation rules [43].

### Promoter sequence analysis

To investigate the promoter regions of ARF genes, 1.5 kb of genomic DNA sequences upstream of initiation codon ATG were retrieved from the genome assembly. *Cis*-regulatory elements (CREs) known to be involved in auxin responses as well as the regulation under biotic and abiotic stresses were selected for examination. Occurrence and distribution of CREs over a given promoter sequence analysis were performed using standard Python scripts. The expected frequency of each motif was calculated using the average G + C content of 28% observed in our chickpea promoters dataset. Probabilities were estimated based on control sets (2000 Monte Carlo simulations, each set *n* = 23 and 1500 bp length). The characterized CREs included: three elements related to dehydration, high salinity and low temperature (MYCATERD1 [CATGTG], MYCATERD22 [CACATG] and ABRE [ACGTGTC]; [45]); three CREs commonly found in defensin promoters (GT1GMSCAM4 [GAAAAA], RAV1AAT [CAACA] and motif CTCTT; [46]); the element AGCBOXNPGLB [AGCCGCC], which is known to be the binding sequence of the ethylene response factor [47]; a pathogen/elicitor response element WBBOXPCWRKY1 [TTTGACY] [48]; a sugar responsive element associated with auxin responses SURE2STPAT21 [AATACAAAA] [49]); and the auxin-responsive element AuxRe1 [TGTCTC] [3].

### Code availability

A custom pipeline of scripts with the open-source interface for the statistics software R, ([50]) and the open-source interface RStudio ([51], http://www.rstudio.com/) were used to retrieve data from NCBI (gene IDs, accession numbers, molecular weights, protein lengths, location on chromosomes and exon counts) and perform data analysis. R markdown and R code files used in this study are available in our git-based, publicly accessible repository (https://github.com/jdieramon/ChickpeaProject). We will continue to update and modify the code repository. However, older versions of the code can be retrieved using the command line-based git program. The code is distributed under the open source MIT License.

## Results and discussion

### Genome-wide identification and chromosomal distribution of *C. arietinum* ARF genes

**Fig. 1:**
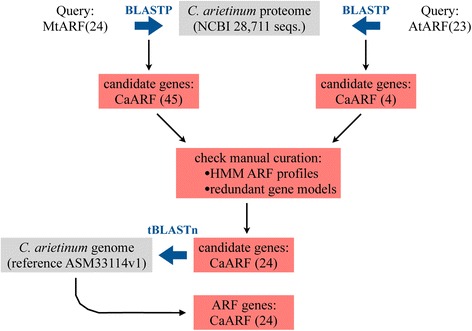
Schema of the workflow to identify ARF genes in *C. arietinum*. *Medicago* ARF proteins were used as query to search for orthologs (BLASTP) in the predicted chickpea genome. The same procedure was applied using *Arabidopsis* ARF proteins. The output was further examined by manual curation and redundant and/or invalid genes were removed from the data set. This step resulted in 24 complete chickpea ARF protein sequences. Those candidates were blasted against the chickpea genome to identify any possible unpredicted gene

The procedure to identify all members of the ARF gene family in the *C. arietinum* genome is shown in Fig. 1. BLASTp searches followed by HMM profile and domain analyses using the NCBI’s CDD tool resulted in the primary identification of 45 potential ARF protein sequences in the chickpea genome. Subsequently, the redundant sequences with the same chromosome locations were removed from the candidate list. In the end, using one gene model per locus, a total of 24 chickpea ARF (CaARF) were extracted and named according to their locations from top to bottom on the chickpea chromosomes (from Chr. 1 to Chr. 8). This nomenclature system is broadly used in genome-wide studies and provides a unique identifier for each member of a given gene family. For example, members of the ARF family have been named that way in rice [52], maize [53], soybean [21] or grape [54]. Information on these 24 sequences (name, locus ID, length, location on chromosome and basic data about the deduced peptide) is listed in Table 1. The exon number of CaARF genes ranged from 2 (*CaARF8*) to 16 (*CaARF12*, *CaARF19*). The sizes of the deduced proteins varied markedly from 444 (*CaARF23*) to 1125 amino acid residues (*CaARF20*). The corresponding molecular masses varied from 50.69 to 126.20 kDa and the predicted isoelectric points (pIs) varied from 5.55 (*CaARF11*) to 8.56 (*CaARF4*). As illustrated by other plant species, the wide range of pIs suggests that the chickpea ARF proteins can work in very different subcellular environments. The percentage of identity between the predicted chickpea and the *Medicago* ARF protein sequences ranged from 40.1% to 94.8% (Table 1). As expected, CaARF were more closely related to those from the model legume *M. truncatula* than those from *Arabidopsis* **(**Additional file 2: Figure S2).

**Table 1:** *ARF* gene family in chickpea

Name ID	Locus ID	Protein ID	Chr	Chr start	Chr end	Strand	Length (aa)	pI	Mol wt. (kDa)	Exons	Isoforms
CaARF1	LOC101492112	XP_004485416	Ca1	154,447	160,822	+	670	5.63	74.764	15	2
CaARF2	LOC101513952	XP_004485844	Ca1	2,832,460	2,837,619	–	711	6.33	77.719	12	3
CaARF3	LOC101501408	XP_004485979	Ca1	3,851,446	3,857,899	–	908	6.17	100.502	14	1
CaARF4	LOC101509547	XP_004487099	Ca1	12,145,610	12,149,407	+	719	8.56	79.314	5	3
CaARF5	LOC101492916	XP_012571810	Ca1	12,333,186	12,338,793	+	826	6.69	92.462	12	1
CaARF6	LOC101498659	XP_004488112	Ca1	24,068,315	24,076,322	–	1120	6.07	125.619	14	3
CaARF7	LOC101504978	XP_004490754	Ca2	28,016,288	28,024,622	–	833	5.89	92.432	14	1
CaARF8	LOC101503141	XP_004490828	Ca2	29,095,001	29,096,913	+	504	6.63	55.295	2	1
CaARF9	LOC101491204	XP_012568938	Ca3	14,022,096	14,027,221	+	671	5.89	75.11	13	1
CaARF10	LOC101505543	XP_004497510	Ca4	20,116,751	20,120,816	–	692	7.05	77.313	4	1
CaARF11	LOC101509304	XP_012570835	Ca4	48,380,932	48,386,526	–	917	5.55	102.376	15	1
CaARF12	LOC101496441	XP_012571326	Ca5	27,748,317	27,758,333	–	853	5.95	94.344	16	2
CaARF13	LOC101504083	XP_004503553	Ca6	4,214,355	4,221,885	+	1120	6.41	123.626	14	2
CaARF14	LOC101498188	XP_004503803	Ca6	6,141,652	6,146,690	–	867	6.09	96.712	14	1
CaARF15	LOC101500671	XP_004504542	Ca6	12,432,295	12,439,057	+	918	6.17	102.686	14	2
CaARF16	LOC101493974	XP_004505103	Ca6	17,365,769	17,371,522	–	725	6.32	80.25	10	2
CaARF17	LOC101505359	XP_012572776	Ca6	27,667,714	27,674,248	–	807	6.53	89.683	12	2
CaARF18	LOC101492451	XP_004506012	Ca6	28,196,797	28,201,150	–	706	7.03	78.446	4	1
CaARF19	LOC101515039	XP_012572936	Ca6	41,935,507	41,941,343	+	691	6.23	76.888	16	1
CaARF20	LOC101489666	XP_004508019	Ca7	3,353,577	3,360,886	–	1125	6.53	126.203	15	7
CaARF21	LOC101514738	XP_004510646	Ca7	34,898,868	34,902,262	+	612	7.61	69.328	4	2
CaARF22	LOC101492136	XP_004510662	Ca7	35,086,399	35,091,870	–	679	6.38	75.972	15	3
CaARF23	LOC101505502	XP_004511136	Ca7	44,457,833	44,469,177	+	444	5.72	50.687	12	1
CaARF24	LOC101514889	XP_012567350	*	*	*	*	598	6.17	65.776	4	1

*CaARF24 was not mapped on any chromosome

**Fig. 2:**
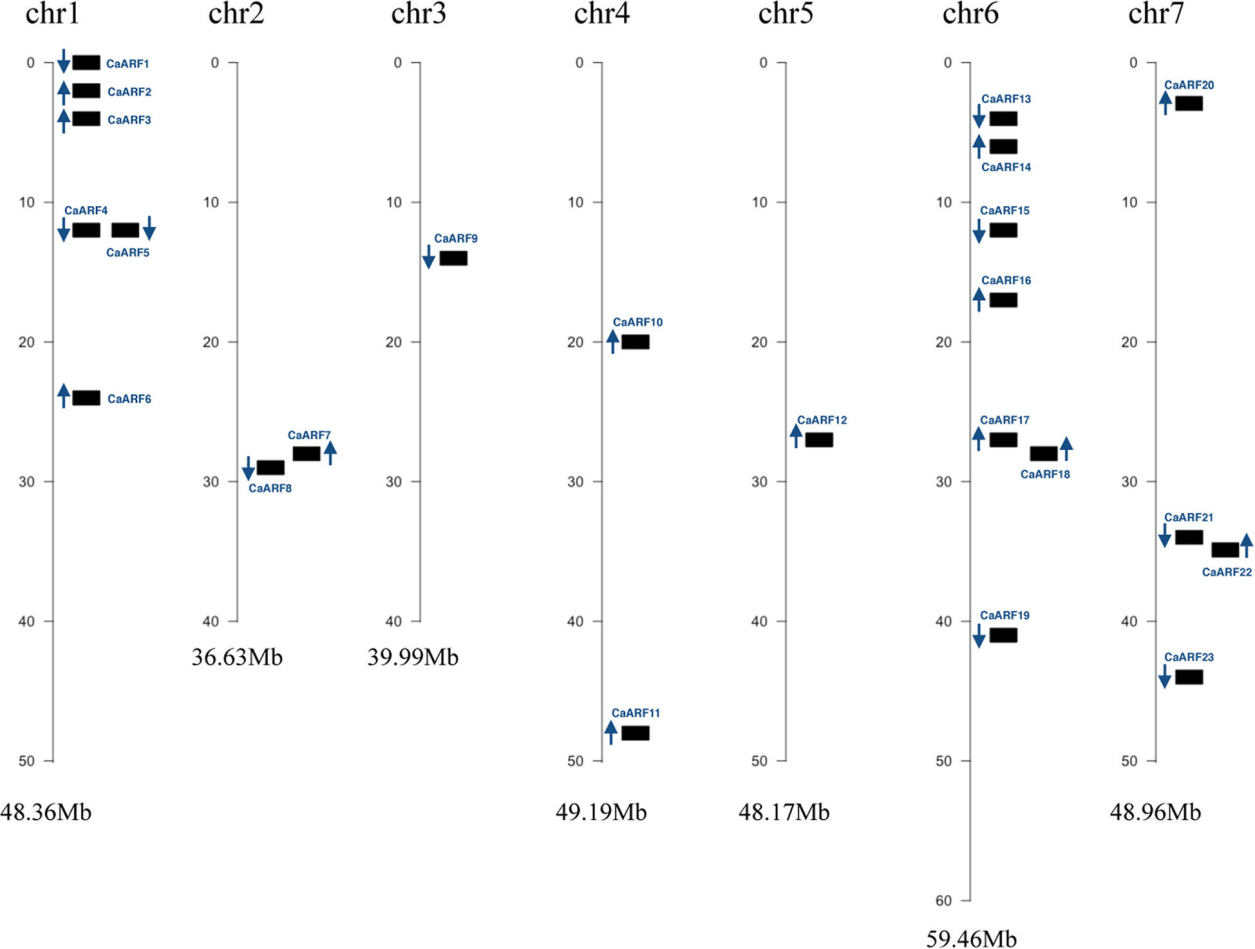
Genomic distribution of CaARF genes on chickpea chromosomes. Only those chromosomes bearing CaARF genes are represented. The chromosome numbers and sizes (Mb) are indicated at the top and bottom of each bar, respectively. The arrows next to gene names show the direction of transcription

CaARF gene locations were mapped on chromosomes in order to gain an insight into the organization of CaARF genes on the genome. Based on the available *C. arietinum* genome assembly, 23 out the 24 CaARF genes were distributed among seven of the eight chromosomes. We could not map *CaARF24*. The other 23 *ARF* genes were unevenly distributed through the chickpea genome. Two chromosomes (chrs. 1 and 6) contained more than 56% of the mapped ARF. The total distribution was the following: Chromosome 6 contained the highest number with 7 ARF genes (30.4%), followed by chromosome 1 (6 genes, 26.1%) and chromosome 7 (4 genes, 17.4%). Chromosome 2 and 4 showed 2 genes each, whereas chromosomes 3 and 1 contained 1 gene each. Chromosome 8, which is the shortest in the chickpea genome, did not contain any ARF gene (Fig. 2 and Additional file 3: Figure S3).

In addition, further investigation showed that two genes (*CaARF15* and *CaARF16*) were located in the vicinity of two QTL clusters (cluster 7 and 8) in chromosome 6 (Linkage Group LG6) associated to drought component traits in chickpea [55]. Those QTLs were located in a consensus map derived from two recombinant inbred line populations**.** Cluster 7, is pointed by the microsatellite marker NCPGR200 (physical position 17,478,000–17,478,700) and comprised some genomic regions associated to days to flowering, days to maturity and harvest index (QR4df02, QR4dm03 and QR4hi02 respectively). Cluster 8, with indicative marker TA106 (12,589,040–12,589,266) included QR3rsa02, QR3pht01 and QR3dm03 for root surface area, plant height and days to maturity respectively. Further studies on these genomic regions will offer insight about the potential role of these ARF for drought tolerance and their value for chickpea breeding.

### Analysis of amino acid composition and classification of CaARF

Next, we aligned and analyzed the amino acid sequences of the CaARF. The domain positions in the 24 CaARF proteins are shown in Additional file 4: Table S1. Sequence analysis of the CaARF proteins, Pfam protein motif- and CDD analyses showed that most of them (18 ARF proteins) harbour the typical ARF structure comprising (1) a highly conserved DNA-binding domain (DBD) in the N-terminal region made of a plant specific B3-type subdomain and an AUX_RESP subdomain, (2) a variable middle region (MR) that functions as an activation or repression domain, and (3) a carboxy-terminal dimerization (CTD) domain similar to that found in Aux/IAA proteins. All proteins with length < 670 aa showed only the DBD domain (B3 and AUX_RESP), whereas all proteins > 725 aa contained the DBD and CTD domains (B3, AUX_RESP and Aux/IAA; Additional file 3: Figure S3). In the canonical ARF structure model, the CTD is made of two highly conserved subdomains (III and IV). Fifteen of those 18 proteins fit into that model, whereas three ARF have a truncated CTD since only subdomain III is present (CaARF4, 10, 18). Six proteins lack domains III and IV.

Then, we analyzed the amino acid composition of the Middle Regions (MRs). Seven proteins harbour a glutamine (Q), serine (S) and leucine (L)-rich middle region implying that these proteins are likely transcriptional activators since glutamine enrichment seems to be a distinctive feature of ARF activators in all plant lineages [4, 6]. The other eleven CaARF proteins containing a CTD domain may function as repressors based on their MRs enriched in serine, proline, glycine and leucine (SPGL). Six CaARF proteins lacking the CTD domain may also be repressors based on their MR amino acid enrichment (Additional file 5: Figure S4 and Additional file 6: Table S2). The activator/repressor ratio among CaARF is 0.41, which is higher than that in *Medicago* (0.26) but lower than *Arabidopsis* (0.59).

It should be noted that the classification of the ARF family into either activators or repressors is based merely on the enrichment of the specific amino acids but it is unclear what mechanisms underlie activation and repression. Therefore, the activator/repressor categorization should be exercised with caution [56].

### Comparative phylogenetic analysis of the ARF family

**Fig. 3:**
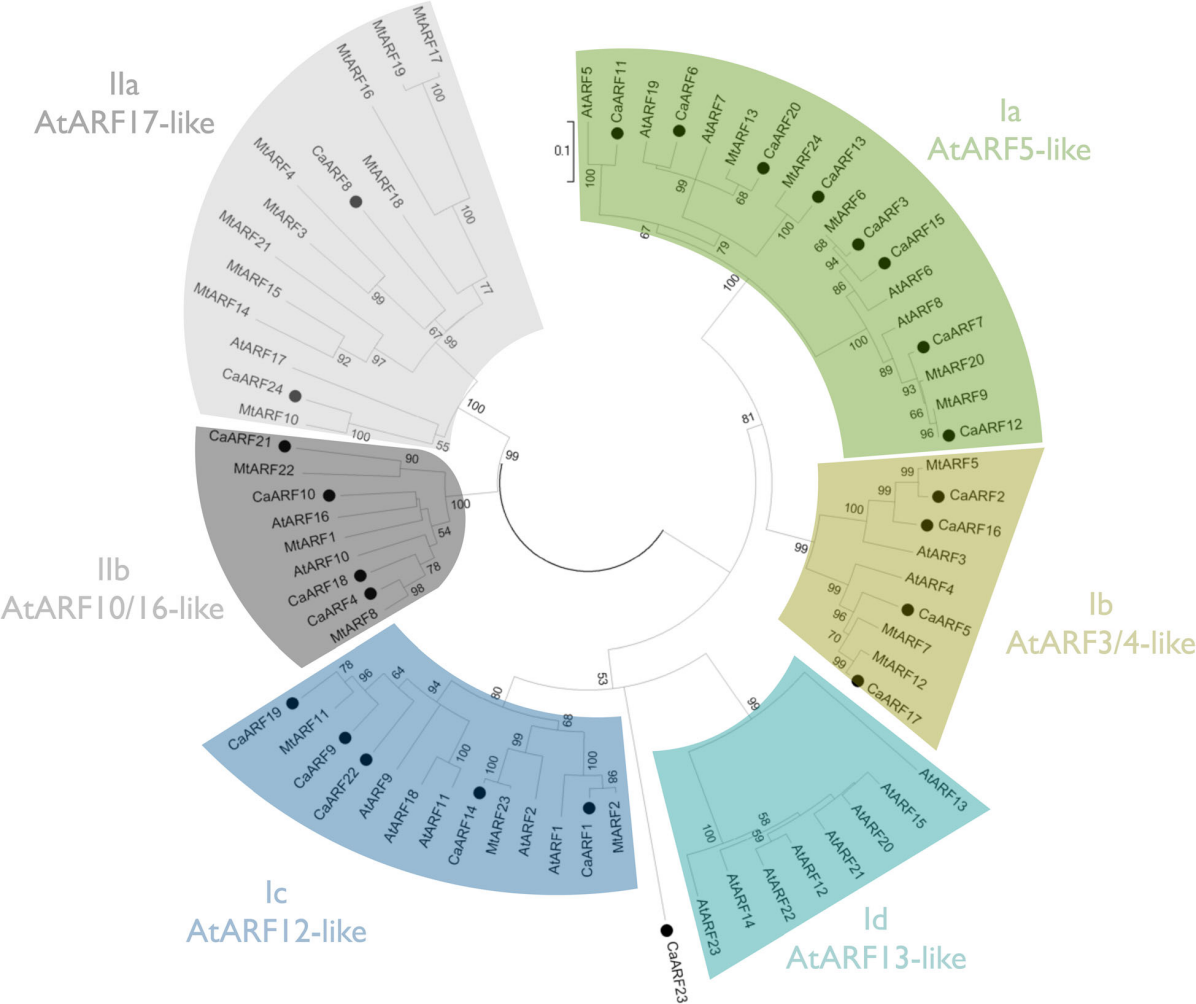
Analysis of phylogenetic relationships of ARF in *C. arietinum*, *M. truncatula* and *A. thaliana*. Twenty-four CaARF proteins, 24 MtARF and 23 AtARF were classified into six subclasses. Scale bar 0.1 denotes the number of amino acid substitutions per site. The percentage of replicate trees in which the associated taxa clustered together in the bootstrap test (1000 replicates) is shown next to the branches. CaARF are marked with solid black circles

To study the phylogenetic relationships between the members of CaARF gene family and to explore the phylogenetic relationships of ARF genes among different species, we used the model plants *Arabidopsis* and *Medicago*. An unrooted tree was constructed from an alignment of 23 AtARF, 24 MtARF and 24 CaARF proteins. The phylogenetic distribution of the 71 ARF revealed that all ARF sequences fall into two major groups (I and II) with well-supported bootstrap values (Fig. 3). Groups II and I are further subdivided into four and two subgroups, respectively. The distribution of each class of CaARF was significantly irregular as the chickpea proteins located on the same chromosome belonged to different groups and subclasses. It has been hypothesized that ARF on the same chromosome may have complementary functions [54]. The group I is the most numerous and contains 18 CaARF, 20 AtARF and 11 MtARF proteins. We labeled as sister pairs those proteins clustered together based on high bootstrap values (> 65%). Related to sister pairs involving chickpea, the group I structures 10 sister pairs (nine pairs of CaARF-MtARF and one pair of CaARF-AtARF). Interestingly, we did not find any sister pair between two chickpea ARF proteins. Chickpea diverged from *M. truncatula* ~ 10–20 million years ago (Mya; [19]). Lack of chickpea sister pairs suggests that recent duplications (after chickpea and *Medicago* separated) have played a very limited role, if any, in the expansion of the ARF chickpea family. All seven potential activator CaARF proteins containing the canonical structure DBD-MR-CTD clustered in subclade Ia. Subclass Id is a lineage-specific clade found in the *Arabidopsis* ARF family. It contains the seven tandem duplicated genes (encoding proteins AtARF12 to ARF15 and AtARF20 to ARF22; [57]) with no homology to any chickpea ARF sequence. Other plant species, such as rice, maize, tomato and grape or Eucalyptus, also lack homologs to that subclass [52, 54, 58–60], implying that these AtARF were derived through a long-term evolution for *Arabidopsis*-specific functions [21]. Between class Ic and Id, an isolated clade clusters CaARF23, with no obvious *Arabidopsis* ortholog. We found that CaARF23 shares 43% identity with the *Eucalyptus grandis* ARF24 at the amino acid level. The EgrARF24 protein has also been clustered in an isolated clade without any *Arabidopsis* ortholog [60]. That clade is absent from the herbaceous annual plants (*Arabidopsis*, tomato and rice) but present in *Eucalyptus* and other woody perennials, so the authors stated that it might be a woody-preferential clade [60]. However, the same authors identified two members of the legume family (*G. max* and *P. vulgaris*) as members of that clade. We found that CaARF23 shared high degree of similarity with those legume proteins (Additional file 7: Figure S5). Therefore, rather than a woody-preferential clade, it is more likely that these are orthologs of an ancestral gene lost in *Arabidopsis* but present in woody perennials as well as some legume species. Finally, group II contains six chickpea ARF members. Three sister pairs (all of them pairs of CaARF-MtARF) were confirmed based on bootstrap values above 90%. Group II also contains the three *Arabidopsis* members (AtARF10, ARF16 and ARF17) that are the most divergent compared with those encoded by the other class [57]. The subclass IIa is made mostly of *Medicago* ARF sequences (10 MtARF, 2 CaARF, 1AtARF) indicating a diverging trend in the evolution of ARF family members across different plant species.

The exon-intron organization of a gene family can provide additional evidences to support the evolutionary relationship among all members. Gene structure tends to remain the same within genes present in the same clade, whereas dissimilarity may be found within clades. The coding sequences of CaARF clustered in group II showed a strong conservation of their gene structure and had an average exon number noticeably lower than those CaARF clustered in group I (3.8 vs. 13.7; t test, *P* < 1e-11). The phenomenon of different exon number has been observed in other species, such as *Arabidopsis*, rice and *B. rapa* [30, 52, 57, 61].

### Diversification of CaARF

We found two major molecular mechanisms underlying the diversification of CaARF proteins, namely diversification by expansion (quantitative diversification) and structural diversification (qualitative diversification). It is important to note that both categories are merely descriptive as they are interconnected and play substantial contribution to the diversity of ARF proteins. For example, genome duplication events are important not only for the numeric expansion of a gene family but also for genomic rearrangements and therefore, for diversification of gene function [6].

#### Diversification by expansion

Duplicated genes represent the source of genetic materials for studying evolution and diversification [62]. We performed duplication analysis using the PGDD software to find the potential relationships between putative paralog pairs of ARF genes and tandem/segmental duplications. ARF genes in tandem clusters have been detected in *Medicago*, *Arabidopsis*, and peach [20, 57, 63].

Chromosomes with more than one ARF gene are candidates to have undergone local gene duplications. We found two genes on chromosome 1 (*ARF4–5*) and two genes on chromosome 7 (*ARF21–22*) that met the criteria to form a cluster as it has been described in the Materials and Methods section. These two pairs are separated by < 190 kb in each case. However, both pairs show relative low values of similarity. *CaARF4* and *CaARF5* show 40% identity at 62% query coverage (Evalue = 9e-81), while *CaARF21* and *CaARF22*, 34% identity at 64% coverage (Evalue = 7e-65). These clusters might have been produced by ancient tandem duplication events. On the other hand, it appears that a number of genes (12 genes, 50%) were segmentally duplicated (Additional file 8: Figure S6). The rate of synonymous substitution per synonymous site (Ks) was used as a proxy for time and the segmental duplications of the CaARF genes were assumed to originate from 48 Mya (million years ago, Ks = 0.59) to 134 Mya (Ks = 1.64). Most of the segmental CaARF duplications seem to have occurred 50–60 Mya. Interestingly, the duplication pattern observed in the CaARF family coincides with the Ks rates found over all the systemic blocks in the chickpea genome that indicates a divergence time of 58 Mya ago [19]. This observation corresponds with the occurrence of whole-genome duplication (WGD) event that occurred at the base of the Papilionoideae (58–60 Mya ago; [64]. Therefore, most of the CaARF duplications originated during the WGD that occurred prior to the speciation of legumes. In order to detect the mode of selection, we evaluated the ratio of nonsynonymous to synonymous nucleotide substitutions (Ka/Ks) among paralogs [65]. Generally, a Ka/Ks ratio > 1 indicates positive selection; a pair of sequences will have a ratio < 1 if one sequence has been under purifying selection, but the other has been drifting neutrally; and a ratio = 1 indicates that both sequences are under neutral evolution [35]. As shown in Additional file 9: Table S3, the average Ka/Ks value of the CaARF gene pairs was 0.19. Most Ka/Ks ratios ranged from 0.11 to 0.27 and none of them was > 1.

These results suggest that essentially, segmental duplications, but not tandem duplications, have contributed to the expansion of the ARF gene family in chickpea. Moreover, the duplicated pair genes have evolved mainly under the influence of purifying selection pressure with no functional divergence after segmental duplications.

#### Structural diversification

Increasing the number of genes within a group through duplication events contribute to the expansion of that group. Another mechanism underlying diversity is domain rearrangements, which involves variations in domain organization. In case of ARF proteins, the alternative domain organizations are illustrated by losses of domains III and/or IV. Numerous and independent losses of domains III and IV seems to have occurred during land plant evolution [6]. However, in spite of these truncations, the proteins are all functional. As said before, six CaARF proteins lack domains III and IV, whereas three ARF have a partial truncated CTD since only subdomain III is present (CaARF4, 10, 18). Therefore, the percentage of CTD-truncated CaARF (37.5%) is more than twice as much as that identified in *Arabidopsis* (17.39%) but lower than in *Medicago* (54%). This may suggest that *C. arietinum* shows a tight auxin-dependent transcriptional regulation, at least compared to *Medicago*. Truncated ARF lack domains of interaction with Aux/IAAs, a sequestration mechanism that is released in the presence of auxin. The truncated ARF are predicted to be unable to interact with Aux/IAA, and hence, they should consequently be insensitive to auxin [66]. Therefore the presence of a large number of CTD-truncated ARF in *Medicago* has been understood as an auxin-independent transcriptional regulation [63]. Nevertheless, the true functional significance of truncations is not well understood and this point remains still open. ARF activators may fit well under the insensitive-to-auxin scenario. However, this hypothesis seems unlikely to be relevant for ARF repressors, which have limited interactions with Aux/IAA proteins [5, 11]. For that reason, it has been pointed out that loss of domains III and IV could also have consequences on the interaction of ARF with other transcription factors [6].

Concerning the protein structure, alternative splicing represents an additional mechanism underlying diversity in ARF proteins. Extensive gene duplication and alternative splicing have generally been viewed as opposite trends in gene family evolution [67, 68]. However, ARF proteins represent a clear example in which both processes play a significant role in functional diversification [6]. Computational survey of the alternative transcripts predicted in the *C. arietinum* genome revealed that at least half of the gene family members display alternative splicing. Seven ARF genes have evidence of two alternative variants, four genes of three variants, and one gene (*CaARF20* on Chr7) shows up to seven different variants. Thus, the number of possible alternative transcripts in the chickpea genome is 45. It would be of interest to further characterize the functional roles of different chickpea isoforms.

Although both, genomic truncations and alternative splicing are diversification mechanisms occurring in a non-preferential chromosome location, we found that they had a differential evolutionary significance. Genomic truncations are mainly restricted to clade II and isolated nodes of subclades Ib and Ic. Alternative splicing, on the contrary, is of widespread occurrence with distribution in branches of subclades Ia, Ib, Ic, and IIb (Additional file 10: Figure S7).

### Gene expression patterns of CaARF

**Fig. 4:**
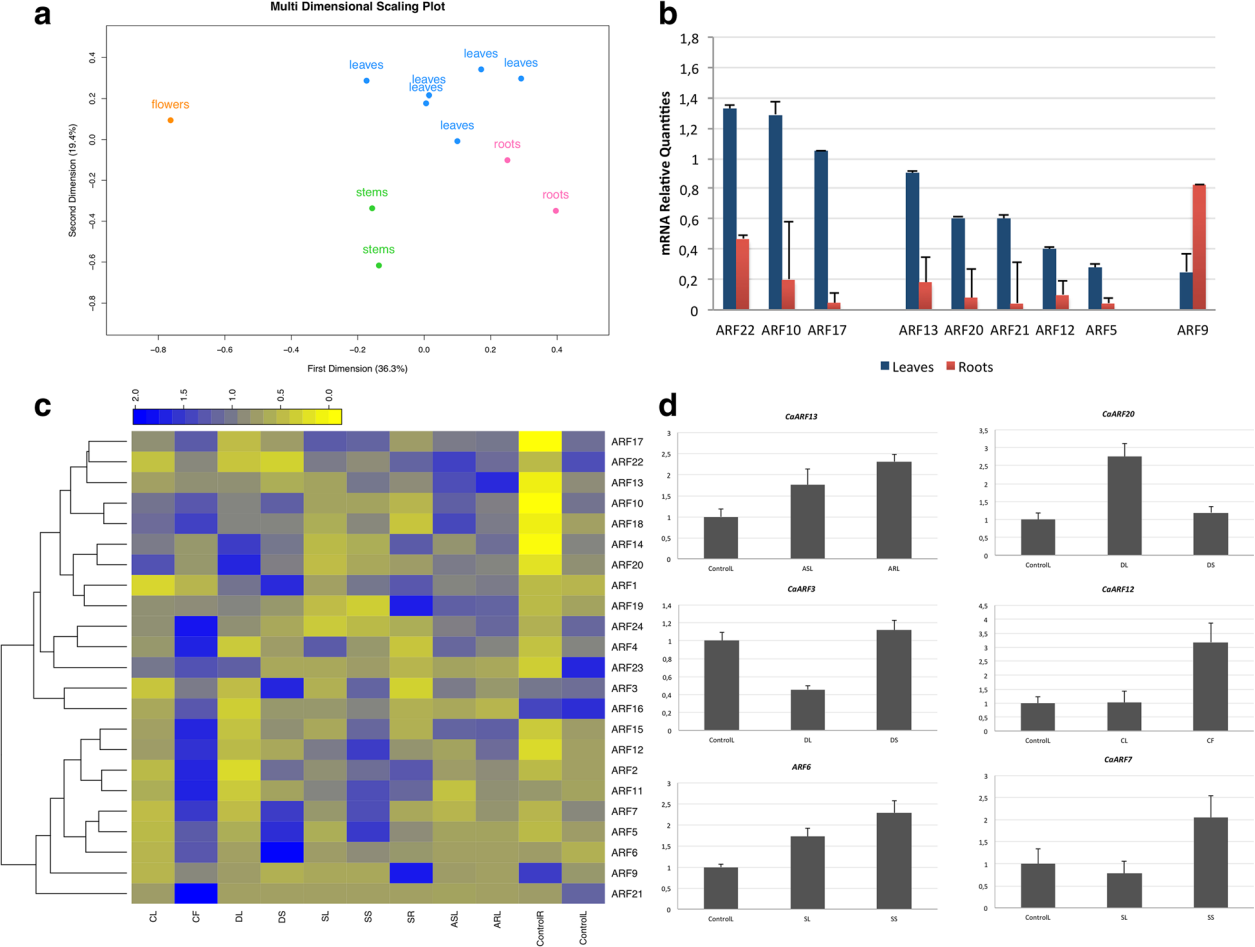
Expression profiles of chickpea ARF genes. Each measurement is the mean of two independent biological samples. **a.** Multidimensional scaling plot showing relationships between sample types. Distance between samples indicates similarity. **b.** Expression levels of ARF genes between leaves and roots. Only those genes showing > 2-fold difference expression are shown. **c.** Hierarchical clustering of CaARF genes. The heat map was constructed using the log2-transformed expression levels. **d.** Expression levels of selected ARF across different samples. NRQs are rescaled to the control sample. Control R, control roots; ControlL, control leaves; ASL, ascochyta susceptible leaves; ARL ascochyta resistant leaves; DL, drought leaves; DS, drought stems; SL, salt leaves; SS, salt stems; SR salt roots; CL, cold leaves; CF, cold flowers

In a first attempt to gain an insight into the putative function of ARF genes in chickpea, we analyzed their expression profiles in different tissues using EST available datasets [69]. Considering the stringent criterion described in the Material and Methods section, nine ARF genes had expression data support (14 ESTs). One ARF (*CaARF23*) hit 3 ESTs, whereas three ARF (*CaARF1*, *14*, *15*) were supported at least by 2 ESTs (Additional file 11: Table S4). Regarding the plant tissues, roots and leaves were the most common hit, although 1 EST from shoot and 1 from embryo were also found. Concerning the experimental conditions, the EST libraries were generated from drought and salinity conditions (11 ESTs in total), during the interaction with the necrotrophic fungus *A. rabiei*, (2 ESTs) and during the embryo development (1 EST). The EST data mining suggests a variety of plant processes and responses to the environment played by ARF. Then, we aimed at measuring the expression levels of the ARF genes in 22 samples representing mainly the tissues and stress conditions showed by the ESTs. The transcript levels for the 24 ARF genes were determined by RT-qPCR. Melting curves showed non-specific PCR product amplification for the *ARF8* primer pair, so that gene was not further considered. We performed singular value decomposition analysis to evaluate the contribution of the stresses and treatments to the variance within the transcript dataset. The largest variance indicated by the first dimension accounts for 35.6% of the total variance in the dataset, while the second dimension accounts for 21.6% of the total variance. This suggests that the dataset contains a moderately high explanatory signal (explaining 57.2%). A multidimensional scaling plot showed that the separation of the samples along the first and second dimension is mainly driven by the tissue (Fig. 4a). Our data support the findings in the model legume *M. truncatula*, whose ARF show complementary tissue-specific auxin responsiveness, with one ARF group induced in shoots and down-regulated in roots, while another group shows the opposite effect [63]. Consistently, when we compared leaves and roots of well-developed plants, we identified a set of ARF expressed at higher levels in leaves than in roots (differences > 2-fold change compared to the reference genes). A second group showed low expression in both, leaves and roots, but the genes were expressed with higher levels in leaves. Finally, *CaARF9* was the only gene that showed higher expression in root tissues vs leaves (Fig. 4b).

The hierarchical clustering of the log2 fold-change values shows that the ARF genes clustered into two main expression groups. A heat map representation (Fig. 4c) indicated that the first group is the largest with 14 genes and contains three predicted activators (*ARF3*, *13*, and *20*). The group is mostly made of genes clustered into clades Ic and IIb (Fig. 3). All genes encoding proteins with a truncated structure but one (*ARF2*), fell into this group. The second group is smaller and contains nine members including the other four activators (*ARF6*, *7*, *12*, and *15*). Most of the genes clustered into clades Ia and Ib fell into this group, which is made of genes encoding proteins with the canonical structure, with the exception of *ARF2*. The heat map representation also indicated that different members display preferences to particular samples. Concerning the first group genes *ARF17*, *22*, *13*, *10*, *18*, *14* and *20* showed extremely low values in well-developed roots but we measured higher levels in roots from plants exposed to salt (*ARF14*, *22*). Some genes in this group also showed increased levels related to controls in leaves inoculated with aschochyta blight (*ARF22*, *13*, *10*, and *18*) or leaves from plants under drought (*ARF20*). Finally, the expression of the predicted activator *ARF3* peaked in stems from drought plants but showed low expression leaves under salinity conditions (Fig. 4d). Genes clustered in the second group are preferentially expressed in flowers and stems. This group is virtually absent from leaves. We only detected high expression levels for *ARF12* and *15* when leaves were from samples infected with ascochyta blight.

### *Cis*-regulatory elements in promoters of CaARF

An important question is how ARF can regulate genes in the context of chromatin. Recently, a chromatin switch mechanism has been proposed to direct ARF-dependent gene activation [70]. Aux/IAA proteins compete with SWI-SNF (SWITCH/SUCROSE NONFERMENTING) recruitment, and thus the Aux/IAA degradation allows chromatin remodeling. This mechanism makes the chromatin region more accessible for other transcription factors [71]. In order to give some insight into the dynamic regulation of ARF we analyzed the occurrence and distribution of *cis*-regulatory elements (CREs) over their promoter sequences. The expected frequency of each CRE was calculated using the average G + C content of 27.8% observed in the chickpea dataset (range 26.8 to 32.2). We screened the proximal and distal regions of promoters (up to 1500 bp upstream of the transcriptional start site, TSS) to identify candidate *cis*-elements that might contribute to the fine regulation of gene expression at the transcriptional level. We were able to retrieve the promoter genomic sequences from the 23 ARF genes that were mapped onto chromosomes. To analyze the promoter regions, first the 1.5 kb of genomic DNA sequences upstream of TSS were used to query the GenBank database (nr) by BLASTx. The results confirmed that these surveyed sequences are not coding sequences. Next, we estimated the number, abundance and position of some CREs that had been associated previously to biotic, abiotic and auxin-dependent responses in the literature. Further statistical analysis indicated that the ARF gene promoters showed an enriched content of the CREs dataset (Table 2). The element CTCTT, which is involved in symbiosis, appeared a total of 77 times in the whole set of 23 promoters (average 3.35 elements/promoter). Motif CTCTT might have an evolutionary conserved function in controlling plant gene expression during the interaction with microbes [48]. The abundance of the element CTCTT in all the chickpea promoters indicates that this element is important for regulation of ARF genes and provides further evidence that auxin signalling plays a central role during plant-microbe interactions [72]. Most of the sequences also contained the frequent elements GT1GMSCAM4 and RAV1AAT (22 promoters each). On the other hand, the AGCBOXNPGLB element was present in only one promoter (*n* = 1). However, this CRE was also clearly overrepresented in our query set. Finally, we tested whether any given CRE is more common in certain promoter regions compared to background. For this, the promoter sequences were divided into 100 bp nucleotide fragments and the content of the 5 CREs most enriched in our dataset was calculated. An increase in density of the fragments was identified at a distance of ~ − 600 to − 400 bp from the TSS, whereas the control set produced CREs with a uniform distribution in the promoter region (Additional file 12: Figure S8). This result indicates that the region of high distribution density (− 600 to − 400) should be considered in the analysis of the auxin responsive regions.

**Table 2:** *Cis*-regulatory elements in chickpea *ARF* promoters

CRE	Motif	Query^a^	Promoters Observed	Total Occurrences Observed^b^	Avg. Number Occurrs. per promoter	Total Occurrs. Expected^c^	Enrichment Factor^d^	*P*-value^e^
AGCBOXNPGLB	AGCCGCC	23	1	1	1.00	0.0575	17.39	0.0041
RAV1AAT	CAACA	23	22	93	4.23	31.02	3.00	0.0337
GT1GMSCAM4	GAAAAA	23	22	72	3.27	28.98	2.48	0.0327
Motif CTCTT	CTCTT	23	23	77	3.35	1.33	2.43	0.0325
SURE2STPAT21	AATACAAAA	23	3	3	1.00	1.58	1.90	0.0194
AuxRe1	TGTCTC	23	6	8	1.33	4.77	1.68	0.0293
WBBOXPCWRKY1	TTTGACY	23	7	8	1.14	1.37	1.41	
MYCATERD22	CACATG	23	6	7	1.17	4.53	1.54	
MYCATERD1	CATGTG	23	7	7	1.00	4.67	1.50	
ABRE	ACGTGTC	23	1	1	1.00	0.69	1.45	

^a^Total number of promoters in the query set

^b^Total number of motifs in the query set

^c^Total number of motifs expected to occur by chance/1.5 kb promoter based on nucleotide frequency in 23 promoter sequences

^d^Number of motifs observed divided by the number of motifs expected to occur by chance

^e^Probabilities based on 2000 Monte Carlo simulations

## Conclusions

In this study we have analyzed the chickpea genome to identify and characterize the ARF gene family by using a broad range of bioinformatic tools. The ARF proteins from *M. truncatula* were retrieved using the Phytozome database. We used NCBI BLAST searches for query of nucleotide and amino acid sequences in the CDC Frontier genome assembly. The HMM profiles were determined through Pfam and CDD databases. The pI was obtained on the ExPASy proteomics server database. Chromosomal locations, locus ID, aa lengths, molecular weights and number of exons were retreived from the NCBI using custom R scripts. Multiple sequence alignment and phylogenetic tree construction were performed using MUSCLE and MEGA, respectively. Exon-intron distributions were analyzed using GSDS server. Gene duplications were determined using the PGDD database and the orthologous relationships were visualized using Circoletto. Amino acid content of the MR domain was calculated using the ‘Biostrings’ Bioconductor package. The putative *cis*-acting regulatory elements in the promoters were analyzed using custom Python scripts. In silico expression data were obtained via the NCBI EST database. The physical map of chromosomal location was generated using the ‘IRanges’ Bioconductor package. The heat map for expression profile was constructed using the ‘Stats’ R package.

Our data suggest that segmental duplications have contributed to the expansion of the ARF gene family in chickpea. The duplicated pairs have evolved mainly under the influence of purifying selection pressure. Genomic truncation and alternative splicing are also important mechanisms for the diversity of the ARF family. Although genomic truncations are restricted to specific clades, alternative splicing shows a widespread distribution. Expression profiles show a close relationship between tissue and expression patterns. Most of the genes from the same phylogenetic class also clustered in one expression branch. This may indicate that ARF genes from the same class perform similar physiological function in chickpea. The expression results give support for various functional roles of ARF genes in a wider range of developmental processes and stresses. Our study also provides a foundation for further comparative genomic analyses and a framework to trace the dynamic evolution of ARF genes on a large time-scale within the Papilionoideae family.

## Additional files

Additional file 1: **Figure S1.** geNorm ranking of 4 reference genes from chickpea samples. The expression stability value (*M*) is shown as bar plot. Vertical numbers at the top indicate the CV values of the reference genes involved in the normalization. The best pair of references (highly stable expression with *M* values < 1 and CV < 0.5) is represented as black bars. (PDF 190 kb)

Additional file 2: **Figure S2.** ARF protein identity between chickpea, *Arabidopsis* and *Medicago*. (PDF 68 kb)

Additional file 3: **Figure S3.** ARF gene family in chickpea. **a.** Distribution of CaARF on chromosomes based on protein length. **b.** HMM profiles of CaARF based on protein length. (PDF 43 kb)

Additional file 4: **Table S1.** Domain positions in 24 CaARF proteins. (PDF 48 kb)

Additional file 5: **Figure S4.** Protein structure of CaARF family. DBD, DNA-binding domain; MR, middle region; CTD, C-terminal dimerization domain; AD, activation domain (orange color); RD, repression domain (green color); Q, glutamine; S, serine; L, leucine; P, proline; G, glycine. (PDF 32 kb)

Additional file 6: **Table S2.** Data of amino acid content in MR domain of CaARF. (PDF 76 kb)

Additional file 7: **Figure S5.** Phylogenetic relationships between the orthologs of CaARF23 in other species. The phylogenetic tree was constructed using the *Arabidopsis* AtARF2 as an outgroup. The species shown in the figure are *Gossypium raimondii* (2), *Theobroma cacao* (1), *Citrus clementine* (1), *Citrus sinensis* (1), *Populus trichocarpa* (2), *Vitis vinifera* (1), *Fragaria vesca* (1), *Prunus persica* (1), *Malus domestica* (2), *Eucalyptus grandis* (1), *Carica papaya* (1), *Phaseolus vulgaris* (1), *Glycine max* (2), and *Aquilegia coerulea* (1). (PDF 55 kb)

Additional file 8: **Figure S6.** Similarity of CaARF genes. Red color shows highest similarity (> 80% identity) followed by orange (70–80%) and green (60–70%) colors. (PDF 12 kb)

Additional file 9: **Table S3.** Duplicated gene pairs of CaARF genes with Ka / Ks values and time of duplication. (PDF 52 kb)

Additional file 10: **Figure S7.** Gene structure and transcripts analyses of ARF members in chickpea. The figure shows members with genomic truncation (losses of domains III and/or IV), and alternative variants. (PDF 111 kb)

Additional file 11: **Table S4.** Tissue distribution profile of chickpea ARF genes according to the number of expressed sequence tags (ESTs) present in NCBI’s EST Database. (PNG 886 kb)

Additional file 12: **Figure S8.** Distribution de CRES. **a.** Simulated data set. **b.** Actual data set. (PDF 47 kb)

## Abbreviations

ARF: Auxin response factor proteins
Aux/IAA: Auxin/Indole-3-Acetic Acid
CDD: Conserved Domain Database
CREs: Cis-regulatory elements
CTD: carboy-terminal dimerization domain
DBD: DNA-binding domain
E: Real-time qPCR amplification efficiency
HMM: Hidden Markov model
Ka: Nonsynonymous substitutions per nonsynonymous site
Ks: Synonymous substitution per synonymous site
MR: Middle region
Mya: Million years ago
pI: Isoelectric point
QTL: Quantitative trait *loci*
RT-qPCR: Reverse transcription quantitative real-time PCR
TSS: Transcriptional start site
WGD: Whole-genome duplication.
