# Supplementary material for: Genome-wide identification of the auxin response factor gene family in Cicer arietinum
Source: BMC Genomics. 2018 Apr 27;19:301. doi: 10.1186/s12864-018-4695-9 (PMC5921756; doi:10.1186/s12864-018-4695-9)
Supplement: Supplementary file 1 — Figure S1. geNorm ranking of 4 reference genes from chickpea samples. The expression stability value (M) is shown as bar plot. Vertical numbers at the top indicate the CV values of the reference genes involved in the normalization. The best pair of references (highly stable expression with M values < 1 and CV < 0.5) is represented as black bars. (PDF 190 kb) [file 12864_2018_4695_MOESM10_ESM.pdf]

Table S2. Data of amino acid content in MR domain of CaARFs.

Gene	Gln (Q)	Ser (S)	Pro (P)	Gly (G)	Leu (L)	Enrichment	HMM
CaARF1	0.03	0.18	0.12	0.11	0.05	SPGL	DBD-MR-CTD
CaARF2	0.06	0.12	0.07	0.13	0.06	SPGL	DBD-MR
CaARF3	0.18	0.12	0.08	0.04	0.1	QSL	DBD-MR-CTD
CaARF4	0.03	0.12	0.07	0.05	0.09	SPGL	DBD-MR-CTD
CaARF5	0.07	0.12	0.05	0.07	0.1	SPGL	DBD-MR-CTD
CaARF6	0.29	0.11	0.09	0.03	0.12	QSL	DBD-MR-CTD
CaARF7	0.18	0.12	0.05	0.1	0.14	QSL	DBD-MR-CTD
CaARF8	0.03	0.18	0.03	0.05	0.08	SPGL	DBD-MR
CaARF9	0.03	0.16	0.05	0.03	0.05	SPGL	DBD-MR-CTD
CaARF10	0.06	0.15	0.1	0.06	0.11	SPGL	DBD-MR-CTD
CaARF11	0.1	0.1	0.1	0.02	0.1	SPGL	DBD-MR-CTD
CaARF12	0.17	0.1	0.08	0.08	0.14	QSL	DBD-MR-CTD
CaARF13	0.19	0.12	0.09	0.03	0.1	QSL	DBD-MR-CTD
CaARF14	0.04	0.17	0.08	0.07	0.07	SPGL	DBD-MR-CTD
CaARF15	0.22	0.11	0.08	0.04	0.09	QSL	DBD-MR-CTD
CaARF16	0.07	0.14	0.06	0.1	0.07	SPGL	DBD-MR
CaARF17	0.06	0.13	0.04	0.09	0.07	SPGL	DBD-MR-CTD
CaARF18	0.06	0.11	0.06	0.07	0.11	SPGL	DBD-MR-CTD
CaARF19	0.05	0.15	0.05	0.03	0.07	SPGL	DBD-MR-CTD
CaARF20	0.27	0.12	0.08	0.03	0.12	QSL	DBD-MR-CTD
CaARF21	0.1	0.15	0.08	0.02	0.09	SPGL	DBD-MR
CaARF22	0.03	0.1	0.08	0.05	0.07	SPGL	DBD-MR-CTD
CaARF23	0	0.5	0	0	0	SPGL	DBD-MR
CaARF24	0.03	0.15	0.03	0.14	0.11	SPGL	DBD-MR
