# Supplementary material for: Genome-wide identification of the auxin response factor gene family in Cicer arietinum
Source: BMC Genomics. 2018 Apr 27;19:301. doi: 10.1186/s12864-018-4695-9 (PMC5921756; doi:10.1186/s12864-018-4695-9)
Supplement: Supplementary file 2 — Figure S2. ARF protein identity between chickpea, Arabidopsis and Medicago. (PDF 68 kb) [file 12864_2018_4695_MOESM11_ESM.pdf]

**Table S3.** Duplicated gene pairs of *CaARF* genes with Ka / Ks values and time of duplication.

| Duplicated gene pair | Ka | Ks | Ka / Ks | Mya (Time) |
|---|---|---|---|---|
| *CaARF2 - CaARF16* | 0.19 | 0.60 | 0.32 | 49.18 |
| *CaARF4 - CaARF10* | 0.33 | 1.64 | 0.20 | 134.43 |
| *CaARF4 - CaARF18* | 0.18 | 0.66 | 0.27 | 54.10 |
| *CaARF5 - CaARF17* | 0.19 | 0.64 | 0.30 | 52.46 |
| *CaARF6 - CaARF20* | 0.09 | 0.76 | 0.12 | 62.30 |
| *CaARF7 - CaARF12* | 0.08 | 0.59 | 0.14 | 48.36 |
| *CaARF9 - CaARF19* | 0.10 | 0.92 | 0.11 | 75.41 |
