# Supplementary material for: Genome-wide identification of the auxin response factor gene family in Cicer arietinum
Source: BMC Genomics. 2018 Apr 27;19:301. doi: 10.1186/s12864-018-4695-9 (PMC5921756; doi:10.1186/s12864-018-4695-9)
Supplement: Supplementary file 3 — Figure S3. ARF gene family in chickpea. a. Distribution of CaARF on chromosomes based on protein length. b. HMM profiles of CaARF based on protein length. (PDF 43 kb) [file 12864_2018_4695_MOESM12_ESM.pdf]

Table S4. Tissue distribution profile of chickpea ARF genes according to the number of expressed sequence tags (ESTs) present in NCBI´s EST Database.

Gene Name	Gene ID	EST tissue	Accession	Genotype	Reference
CaARF1	LOC101492112	root	HO067475	RIL from the cross ICC4958xICC1882	[1]
CaARF1	LOC101492112	leaf	FE670495	XJ-209	[2]
CaARF2	LOC101513952	leaf	FE670500	XJ-209	[2]
CaARF3	LOC101501408	root	GR403988	ICCV2	[3]
CaARF7	LOC101504978	leaf	ES560336	Pusa-362	[4]
CaARF12	LOC101496441	leaf	ES560336	Pusa-362	[4]
CaARF14	LOC101498188	shoot	HO066200	ICC4958	[1]
CaARF14	LOC101498188	embryo	JK709419	ICCV2	[5]
CaARF15	LOC101500671	leaf	FE671045	XJ-209	[2]
CaARF15	LOC101500671	root	GR403988	ICCV2	[3]
CaARF20	LOC101489666	root	HO064721	ICC4958	[1]
CaARF23	LOC101505502	root	HO064459	ICC4958	[1]
CaARF23	LOC101505502	root	HO062185	ICC4958	[1]
CaARF23	LOC101505502	root	GR399001	ICC4958	[3]

[1] Deokar, A.A., Kondawar, V., Jain, P.K. et al. Comparative analysis of expressed sequence tags (ESTs) between drought-tolerant and -susceptible genotypes of chickpea under terminal drought stress. BMC Plant Biology (2011) 11:70

[2] Gao, W.R., Wang, X-S., Liu, Q.Y. et al. Comparative analysis of ESTs in response to drought stress in chickpea (*C. arietinum* L.). Biochem Biophysical Res Comm (2008) 376:578-583

[3] Varshney RK, Hiremath PJ, Lekha P, et al. A comprehensive resource of drought- and salinity-responsive ESTs for gene discovery and marker development in chickpea (*Cicer arietinum* L.). BMC Genomics (2009) 10:523

[4] Jaiswal, P., Cheruku, J.R., Kumar, K. et al. Differential transcript accumulation in chickpea during early phases of compatible interaction with a necrotrophic fungus *Ascochyta rabiei.* Mol Biol Rep (2012) 39: 4635.

[5] Gupta S, Garg V, Bhatia S (2015) A New Set of ESTs from Chickpea (*Cicer arietinum* L.) Embryo Reveals Two Novel F-Box Genes, *CarF-box_PP2* and *CarF-box_LysM*, with Potential Roles in Seed Development. PLoS ONE 10(3): e0121100.
