# Supplementary figures and images for: Genome-wide identification of the auxin response factor gene family in Cicer arietinum
Source: BMC Genomics. 2018 Apr 27;19:301. doi: 10.1186/s12864-018-4695-9 (PMC5921756; doi:10.1186/s12864-018-4695-9)

Biological rep. 1

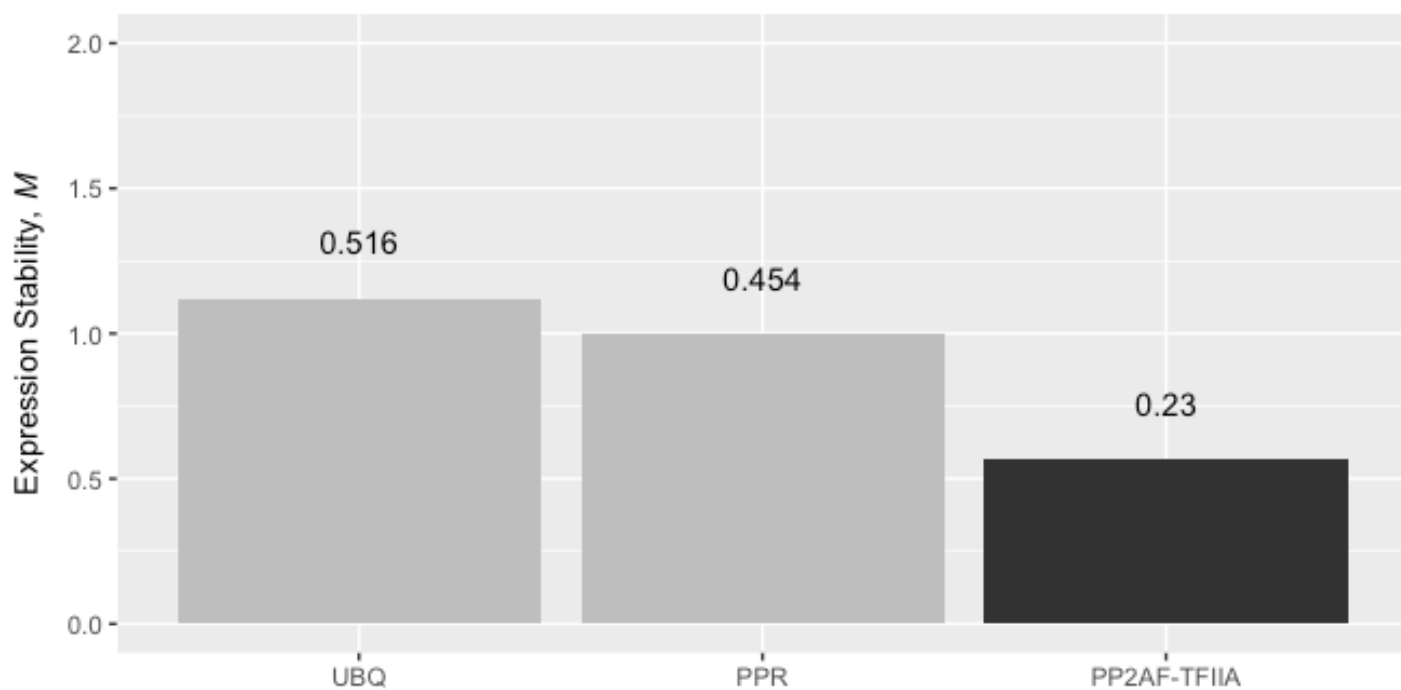

Biological rep. 2

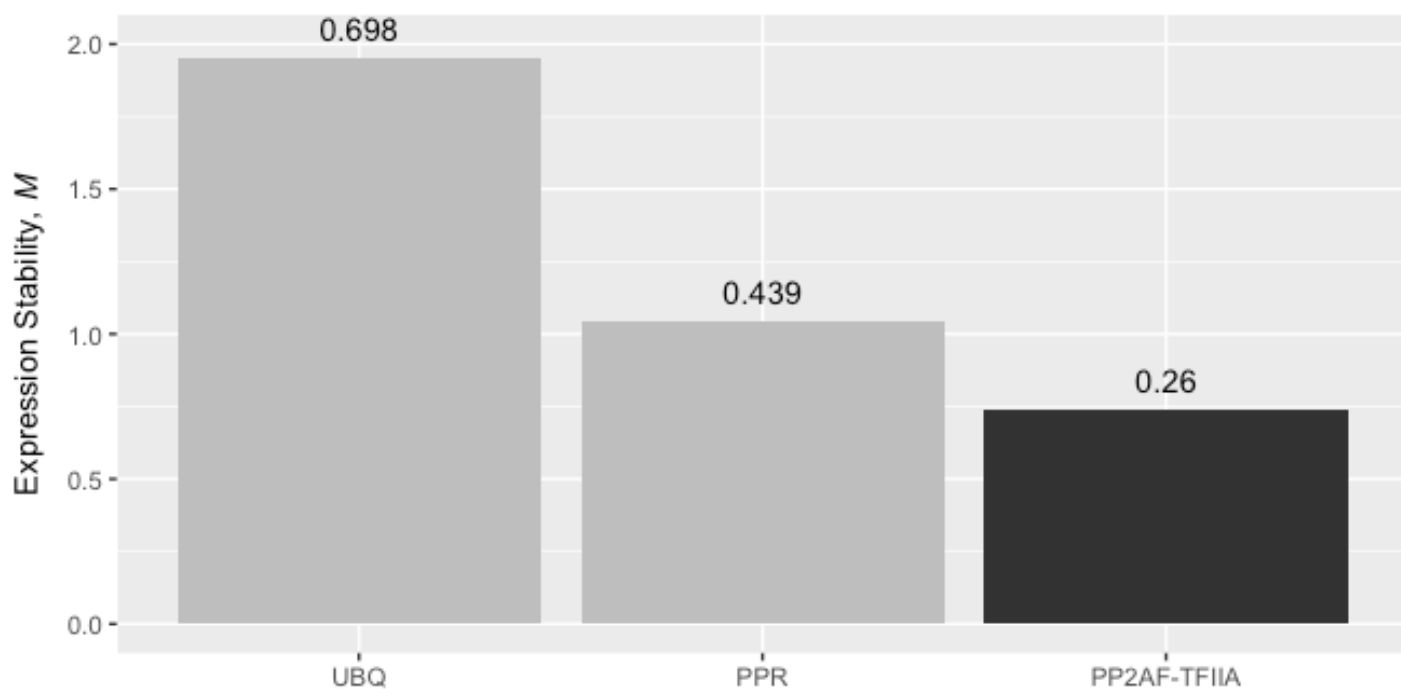

Supplement: Supplementary file 4 — Table S1. Domain positions in 24 CaARF proteins. (PDF 48 kb) [file 12864_2018_4695_MOESM1_ESM.pdf]

Identity between CaARFs and ARFs from model species

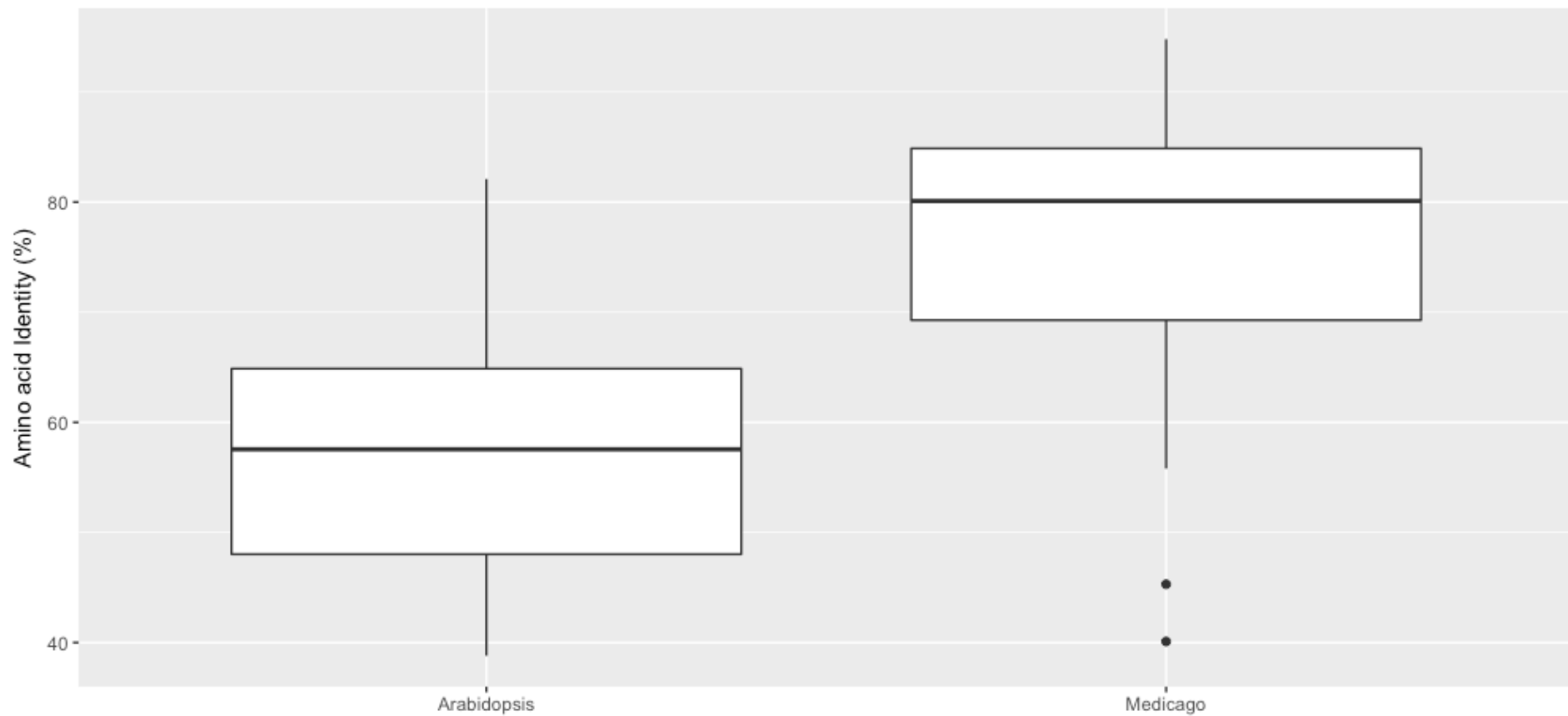

Supplement: Supplementary file 5 — Figure S4. Protein structure of CaARF family. DBD, DNA-binding domain; MR, middle region; CTD, C-terminal dimerization domain; AD, activation domain (orange color); RD, repression domain (green color); Q, glutamine; S, serine; L, leucine; P, proline; G, glycine. (PDF 32 kb) [file 12864_2018_4695_MOESM2_ESM.pdf]

CaARF3, 6, 7, 12, 13, 15, 20

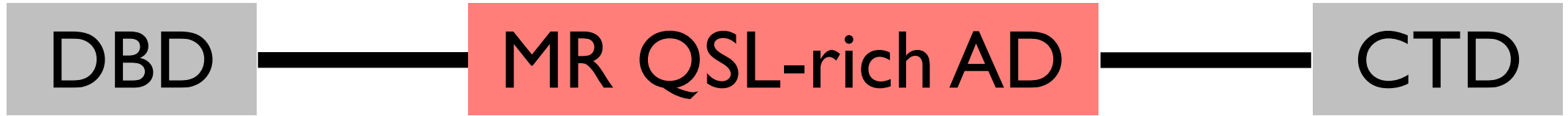

CaARF1, 4, 5, 9, 10, 11, 14, 17, 18, 19, 22

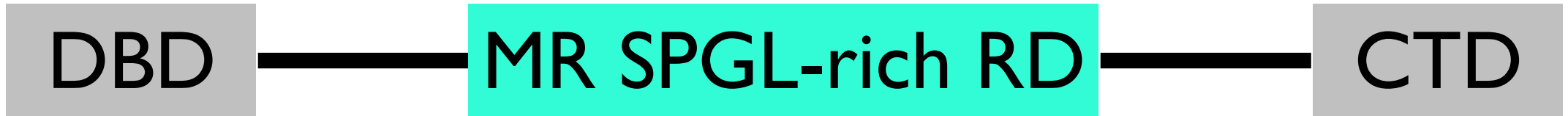

CaARF2, 8, 16, 21, 23, 24

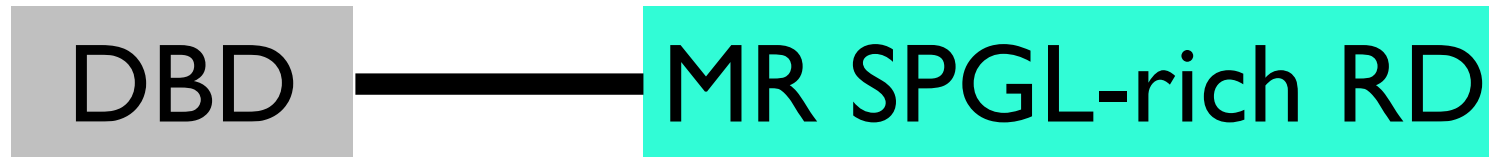

Supplement: Supplementary file 7 — Figure S5. Phylogenetic relationships between the orthologs of CaARF23 in other species. The phylogenetic tree was constructed using the Arabidopsis AtARF2 as an outgroup. The species shown in the figure are Gossypium raimondii (2), Theobroma cacao (1), Citrus clementine (1), Citrus sinensis (1), Populus trichocarpa (2), Vitis vinifera (1), Fragaria vesca (1), Prunus persica (1), Malus domestica (2), Eucalyptus grandis (1), Carica papaya (1), Phaseolus vulgaris (1), Glycine max (2), and Aquilegia coerulea (1). (PDF 55 kb) [file 12864_2018_4695_MOESM4_ESM.pdf]

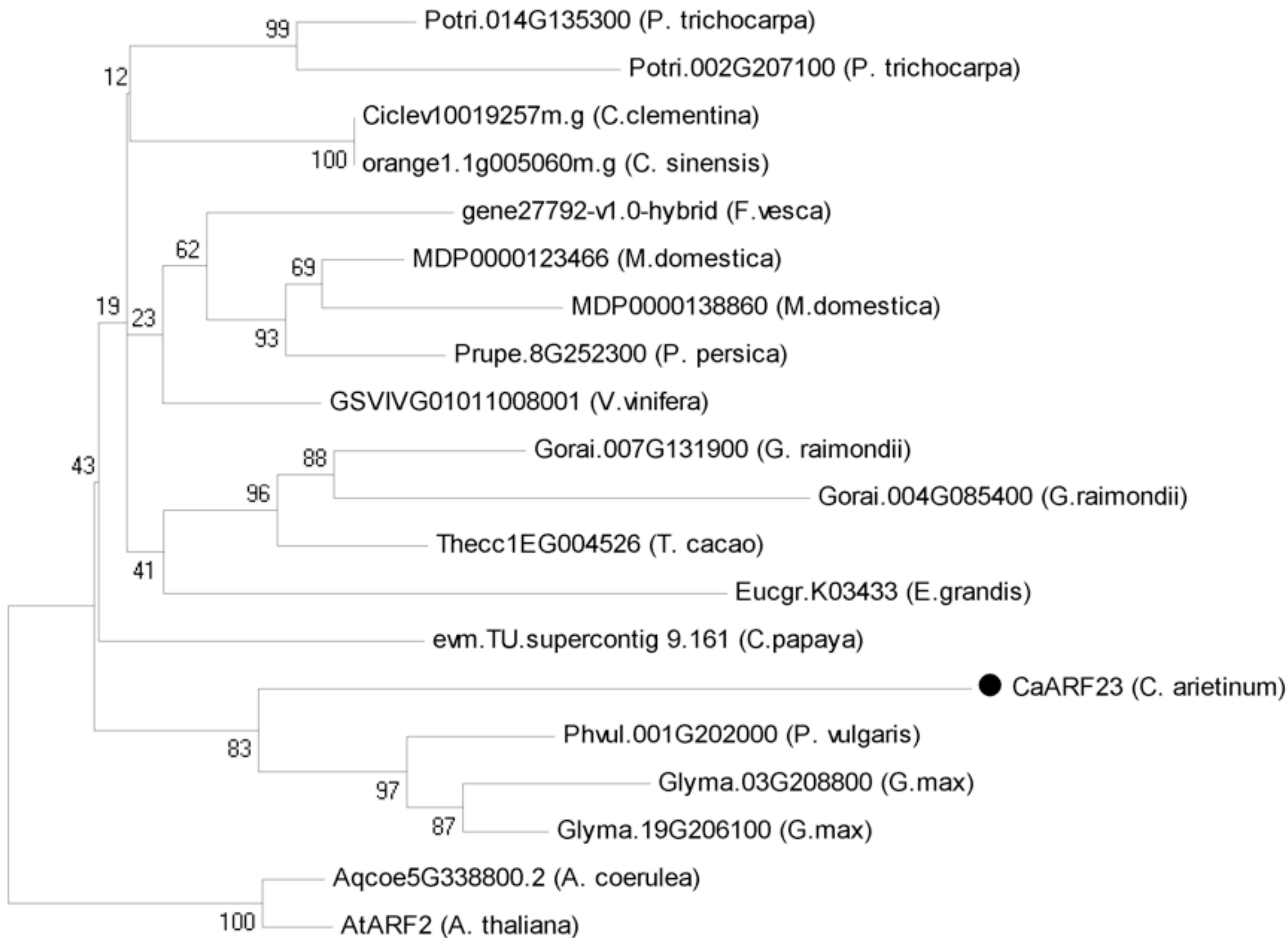

Supplement: Supplementary file 8 — Figure S6. Similarity of CaARF genes. Red color shows highest similarity (> 80% identity) followed by orange (70–80%) and green (60–70%) colors. (PDF 12 kb) [file 12864_2018_4695_MOESM5_ESM.pdf]

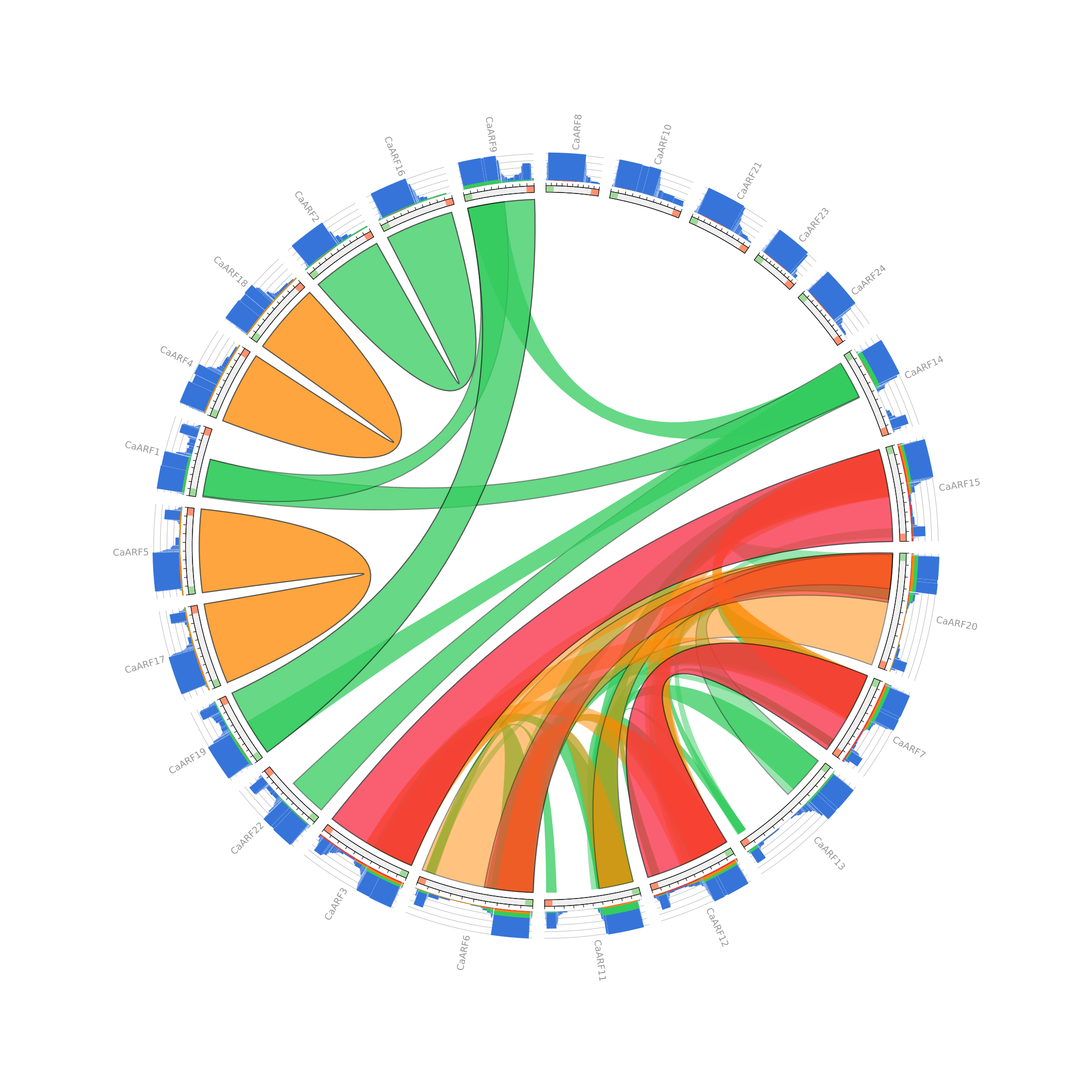

Supplement: Supplementary file 9 — Table S3. Duplicated gene pairs of CaARF genes with Ka / Ks values and time of duplication. (PDF 52 kb) [file 12864_2018_4695_MOESM6_ESM.pdf]

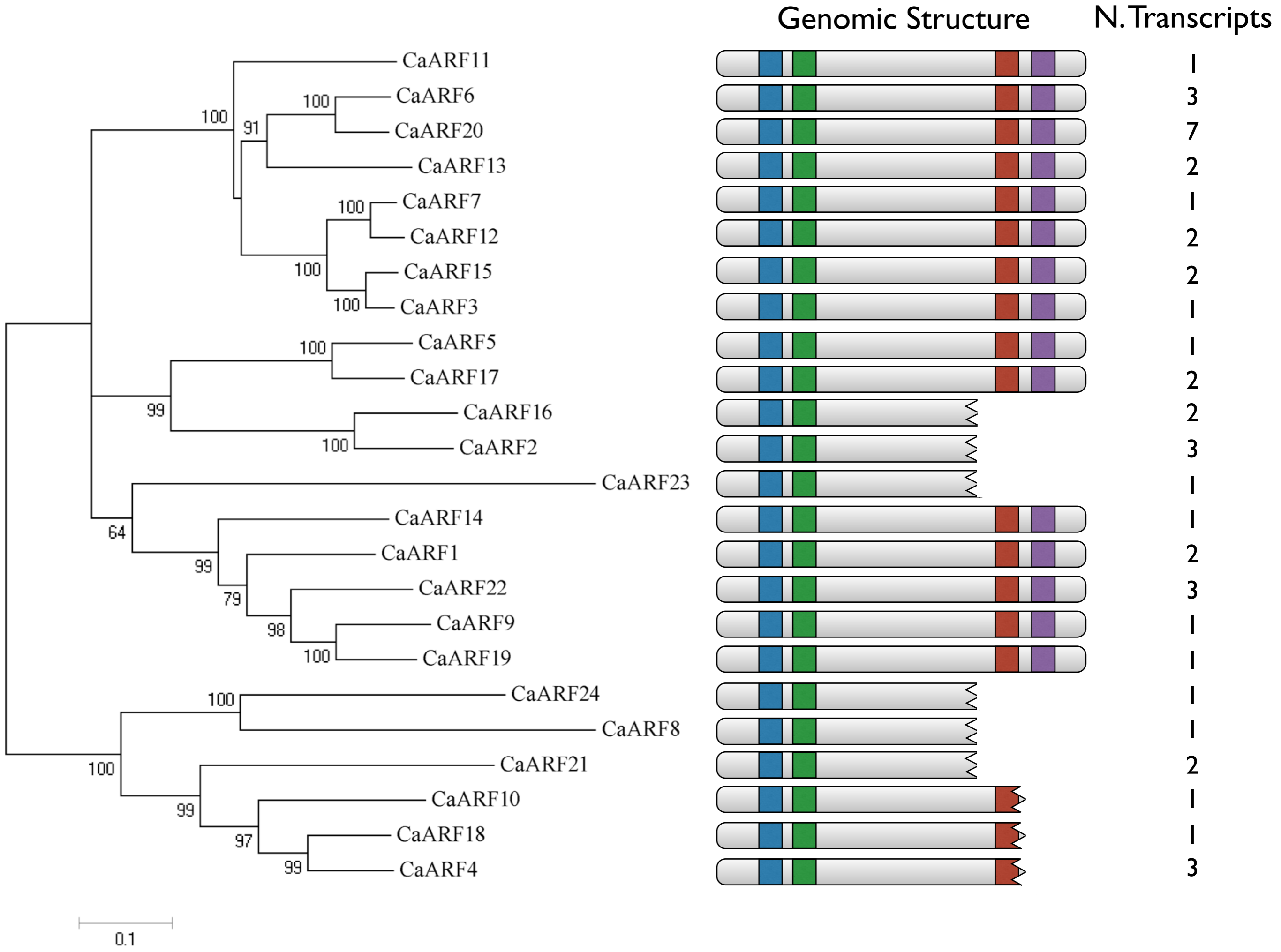

Supplement: Supplementary file 10 — Figure S7. Gene structure and transcripts analyses of ARF members in chickpea. The figure shows members with genomic truncation (losses of domains III and/or IV), and alternative variants. (PDF 111 kb) [file 12864_2018_4695_MOESM7_ESM.pdf]

**a**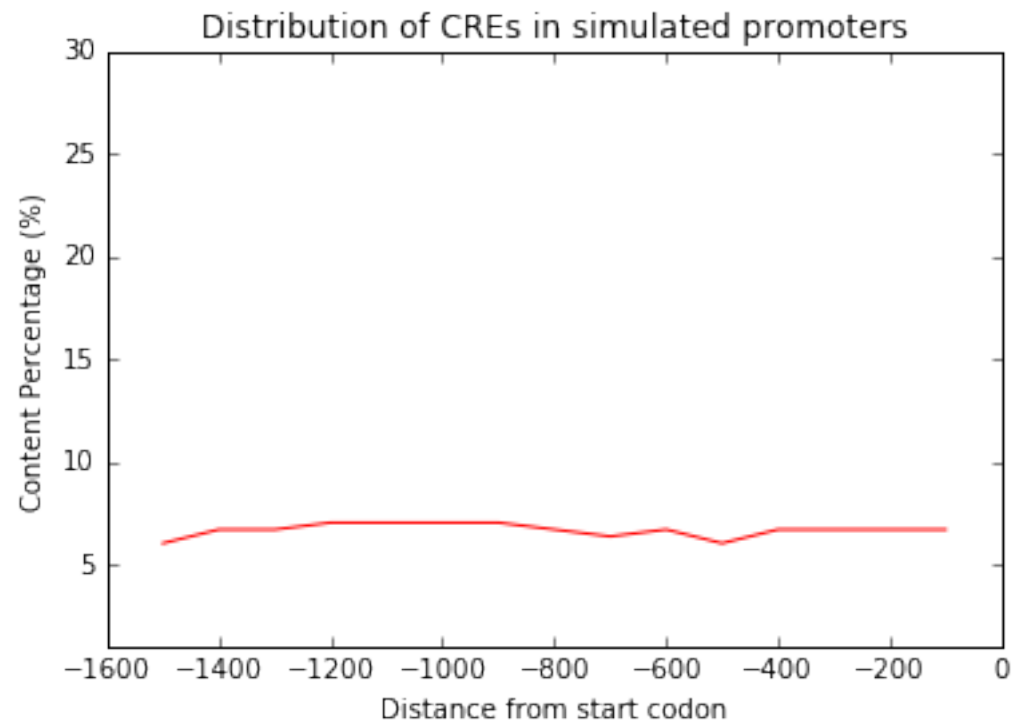**b**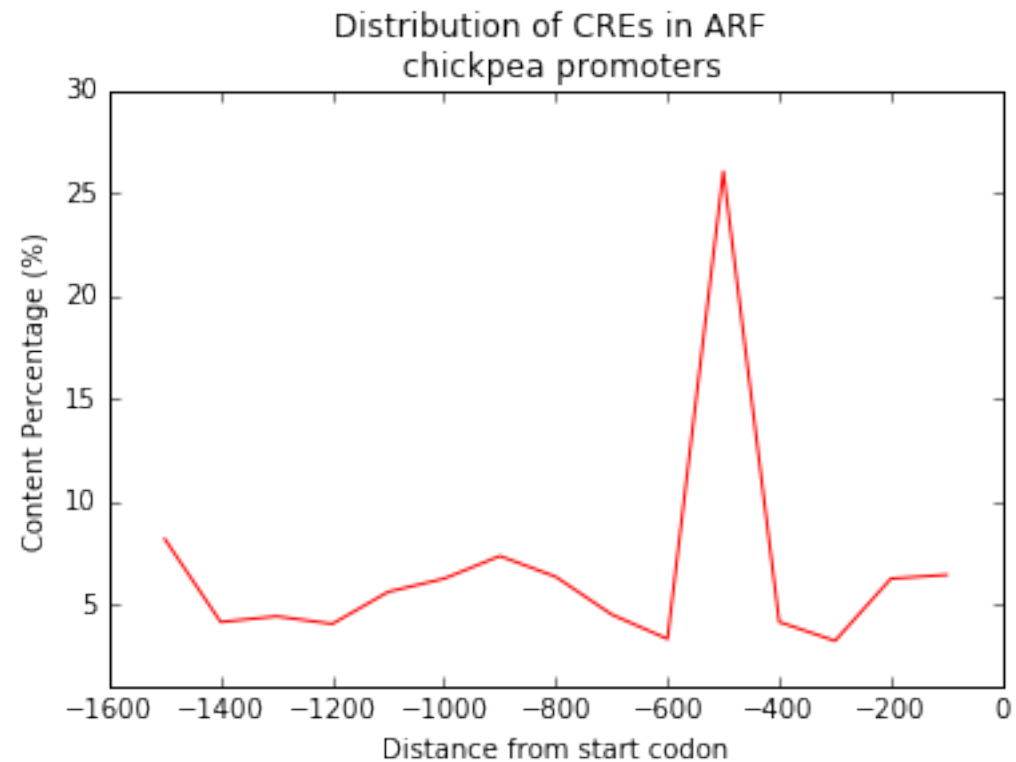

Supplement: Supplementary file 11 — Table S4. Tissue distribution profile of chickpea ARF genes according to the number of expressed sequence tags (ESTs) present in NCBI’s EST Database. (PNG 886 kb) [file 12864_2018_4695_MOESM8_ESM.png]
