# Supplementary material for: Genome-wide identification of the auxin response factor gene family in Cicer arietinum
Source: BMC Genomics. 2018 Apr 27;19:301. doi: 10.1186/s12864-018-4695-9 (PMC5921756; doi:10.1186/s12864-018-4695-9)
Supplement: Supplementary file 6 — Table S2. Data of amino acid content in MR domain of CaARF. (PDF 76 kb) [file 12864_2018_4695_MOESM3_ESM.pdf]

a

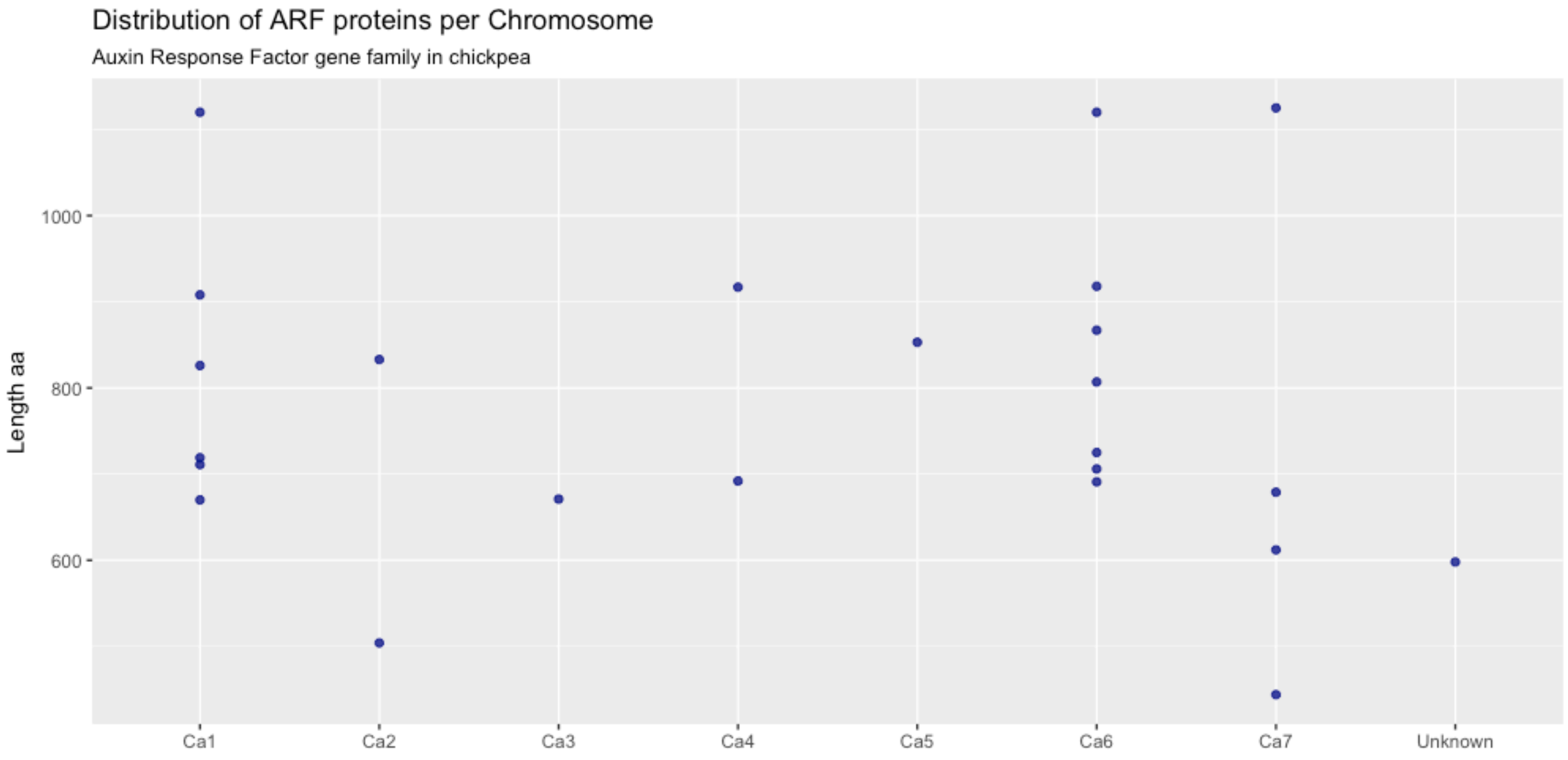

Distribution of ARF proteins per Chromosome
Auxin Response Factor gene family in chickpea
Length aa
1000
800
600
Ca1
Ca2
Ca3
Ca4
Ca5
Ca6
Ca7
Unknown

b

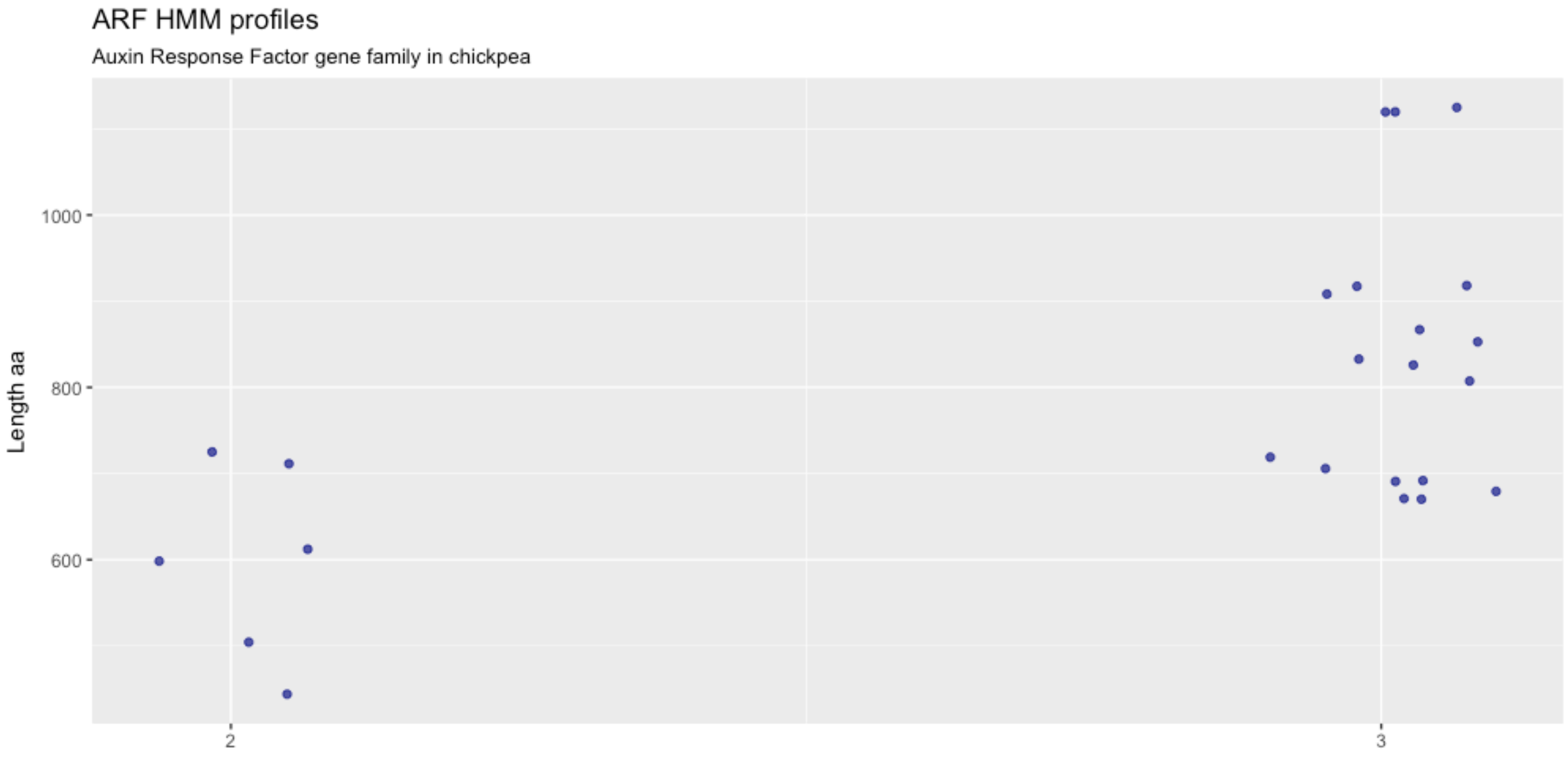

ARF HMM profiles
Auxin Response Factor gene family in chickpea
Length aa
1000
800
600
2
3
