# Supplementary material for: Genome-wide identification of the auxin response factor gene family in Cicer arietinum
Source: BMC Genomics. 2018 Apr 27;19:301. doi: 10.1186/s12864-018-4695-9 (PMC5921756; doi:10.1186/s12864-018-4695-9)
Supplement: Supplementary file 12 — Figure S8. Distribution de CRES. a. Simulated data set. b. Actual data set. (PDF 47 kb) [file 12864_2018_4695_MOESM9_ESM.pdf]

Table S1. Domain positions in 24 CaARF proteins.

RefSeq protein	Gene ID	Gene name	Length (aa)	DBD (aa)	MR (aa)	CTD (aa)
XP_004485416	LOC101492112	*CaARF1*	670	21-375	376-470	471-635
XP_004485844	LOC101513952	*CaARF2*	711	46-403	404-568	None[1]
XP_004485979	LOC101501408	*CaARF3*	908	22-379	380-657	658-864
XP_004487099	LOC101509547	*CaARF4*	719	10-412	413-546	547-692[2]
XP_012571810	LOC101492916	*CaARF5*	826	62-426	427-624	625-787
XP_004488112	LOC101498659	*CaARF6*	1120	22-377	378-815	816-1082
XP_004490754	LOC101504978	*CaARF7*	833	23-380	381-601	602-796
XP_004490828	LOC101503141	*CaARF8*	504	11-366	367-405	None[1]
XP_012568938	LOC101491204	*CaARF9*	671	13-368	369-496	497-649
XP_004497510	LOC101505543	*CaARF10*	692	22-423	424-543	544-684[2]
XP_012570835	LOC101509304	*CaARF11*	917	44-399	400-681	682-900
XP_012571326	LOC101496441	*CaARF12*	853	25-382	381-620	621-815
XP_004503553	LOC101504083	*CaARF13*	1120	35-392	393-788	789-1097
XP_004503803	LOC101498188	*CaARF14*	867	62-417	418-561	562-833
XP_004504542	LOC101500671	*CaARF15*	918	22-378	379-672	673-874
XP_004505103	LOC101493974	*CaARF16*	725	41-400	401-561	None[1]
XP_012572776	LOC101505359	*CaARF17*	807	59-424	425-605	606-768
XP_004506012	LOC101492451	*CaARF18*	706	11-406	407-532	533-679[2]
XP_012572936	LOC101515039	*CaARF19*	691	21-377	378-500	501-667
XP_004508019	LOC101489666	*CaARF20*	1125	22-377	378-824	825-1087
XP_004510646	LOC101514738	*CaARF21*	612	30-419	420-524	None[1]
XP_004510662	LOC101492136	*CaARF22*	679	27-380	381-496	497-648
XP_004511136	LOC101505502	*CaARF23*	444	21-359	360-361	None[1]
XP_012567350	LOC101514889	*CaARF24*	598	18-400	401-488	None[1]

[1] Without domain III, IV : 2,8,16,21,23,24

[2] Without domain IV: 4,10,18
